# Topological Methods for Studying Contextuality: *N*-Cycle Scenarios and Beyond

**DOI:** 10.3390/e25081127

**Published:** 2023-07-27

**Authors:** Aziz Kharoof, Selman Ipek, Cihan Okay

**Affiliations:** Mathematics Department, Bilkent University, Ankara 06800, Türkiye; aziz.kharoof@bilkent.edu.tr (A.K.); selman.ipek@bilkent.edu.tr (S.I.)

**Keywords:** quantum information, quantum contextuality, quantum nonlocality, algebraic topology

## Abstract

Simplicial distributions are combinatorial models describing distributions on spaces of measurements and outcomes that generalize nonsignaling distributions on contextuality scenarios. This paper studies simplicial distributions on two-dimensional measurement spaces by introducing new topological methods. Two key ingredients are a geometric interpretation of Fourier–Motzkin elimination and a technique based on the collapsing of measurement spaces. Using the first one, we provide a new proof of Fine’s theorem characterizing noncontextual distributions in *N*-cycle scenarios. Our approach goes beyond these scenarios and can describe noncontextual distributions in scenarios obtained by gluing cycle scenarios of various sizes. The second technique is used for detecting contextual vertices and deriving new Bell inequalities. Combined with these methods, we explore a monoid structure on simplicial distributions.

## 1. Introduction

Quantum contextuality is a fundamental feature of collections of probability distributions obtained from quantum measurements. In a classical setting, experimental statistics are derivable from a joint probability distribution. Measurements of quantum observables, however, do not satisfy this principle, leading to violations of Bell inequalities, or more generally, noncontextual inequalities, which serve as a witness of this quintessentially nonclassical phenomenon. That such violations were *necessary* was first discovered by Bell [1]. Later, Fine [2,3] showed that such inequalities were also *sufficient* for recovering a classical description in the well-known Clauser–Horne–Shimony–Holt (CHSH) scenario [4].

A systematic study of contextuality scenarios using sheaf theory was introduced by Abramsky–Brandenburger in [5]. Later topological ideas from group cohomology were introduced to the study of contextuality [6], with an emphasis on investigating quantum advantage in measurement-based quantum computation. More recently, a unified framework for the study of contextuality was introduced, based on combinatorial representations of topological spaces known as simplicial sets [7]. The basic objects in this theory are called simplicial distributions. This theory subsumes the theory of nonsignaling distributions and goes beyond by formulating the notion of distributions on spaces rather than sets. Contextuality can be formulated in this generality.

**Figure 1:**
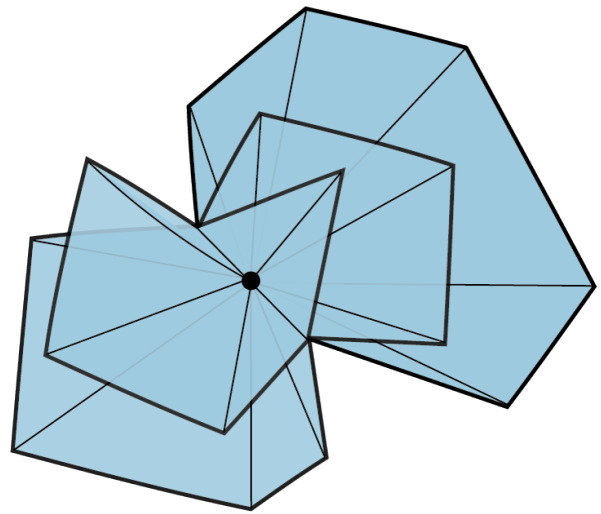
Flower scenario.

Initial applications of simplicial distributions in [7] included a new topological proof of Fine’s theorem for the CHSH scenario. A novel feature of this approach is its flexibility in realizing measurement scenarios as topological spaces. Such expressiveness allows for contextuality to be characterized topologically in multiple ways. For instance, one realization of the CHSH scenario is topologically equivalent to a disk consisting of four triangles, while another realization, also appearing in [8], is given by a punctured torus. While the former allows for an analysis similar in spirit to that of Fine, the latter work supplies an alternative proof by the classifying the extreme distributions on the torus. In this paper, we go beyond these examples and consider a generalization of *N*-cycle scenarios [9,10] which we call *flower scenarios*. The flower scenario is obtained by gluing various cycle scenarios of arbitrary size, as in Figure 1. This scenario is a particular example of a class of two-dimensional measurement spaces. Given a one-dimensional simplicial set, i.e., a graph, the cone construction produces a two-dimensional simplicial set. This construction introduces a new vertex and a set of triangles connecting each edge on the graph to the new vertex. For a one-dimensional space *X*, we will write C(X) for the cone space. We will write $L_{N}$ for the space obtained by gluing *N* edges in the shape on a line.

**Theorem** **1.** *Let C(X) denote the flower scenario (Figure 1), the cone of X obtained by gluing the lines $L_{N_{1}},\cdots ,L_{N_{k}}$ at their end points. A simplicial distribution p∈sDist(C(X)) is noncontextual if and only if for every N-circle C on X, the restriction ${p|}_{C}$ satisfies the N-circle inequalities (in the literature, what we refer to as N-circle inequalities are known as N-cycle inequalities. We diverge in terminology by emphasizing the underlying topological space, which is a circle).*

**Figure 8:**
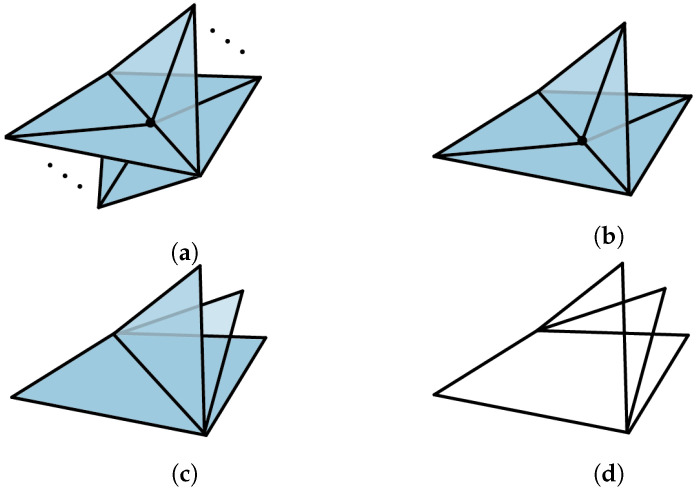
(**a**) Topological realization of the (2,m,2,2) Bell scenario. (**b**) Measurement space for the (2,3,2,2) Bell scenario. (**c**) The bouquet of classical *N*-disks (here, all 3-disks) used in proof of Fine’s theorem for the (2,3,2,2) Bell scenario. (**d**) A distribution on the (2,3,2,2) Bell scenario is classical if and only if 6=42 sets of CHSH inequalities are satisfied, corresponding to the 6 possible circles.

The primary technique that goes into the proof of this result is the Fourier–Motzkin (FM) elimination [11], a version of Gaussian elimination for inequalities. In Section 3.1 we present a topological interpretation of FM elimination. A measurement space is represented by a simplicial set whose simplices correspond to measurements. In this paper, we will restrict our attention to two-dimensional simplicial sets, that is, those obtained by gluing triangles. Our outcome space will be fixed to a canonical choice obtained from $Z_{2}=\{0,1\}$ (known as the nerve space) so that the measurements labeling the edges have binary outcomes. In this setting, noncontextuality is characterized by Bell inequalities consisting of variables corresponding to probabilities of measurements on the edges. For our topological proof of Fine’s theorem, we consider a particular triangulation of the disk, which we refer to as a *classical N-disk*. On these disks, any simplicial distribution turns out to be noncontextual, hence the name classical. If we start from a distribution on the boundary of a disk, the *N*-circle inequalities appear as the sufficient and necessary condition for extending such a distribution from the boundary to the entire disk (Proposition 9). Now, given two such classical disks glued at a common edge, the topological interpretation of FM elimination is that the boundary of the new space is formed by taking the union of the boundaries of the disks and omitting the common edge; see Figure 8. The elimination of the edge is the geometric interpretation of removing the variable by FM elimination. This key idea allows us to characterize the extension condition from the boundary of a bouquet of classical disks, i.e., a collection of disks glued at a common edge, by a collection of circle inequalities (Corollary 5). This extension result is the main ingredient of the proof of Theorem 2 that characterizes noncontextual distributions in the flower scenario Figure 1. Note that this scenario generalizes bipartite Bell scenarios where Alice performs 2 measurements, and Bob performs *m* measurements, and all measurements have binary outcomes [12].

Our next main contribution is the collapsing of measurement spaces to detect contextual vertices of simplicial distributions (Section 5). To study simplicial distributions, on the cone space we introduce a technique based on collapsing edges. Let π:X→X/σ denote the map that collapses an edge σ of the graph. Applying the cone construction gives a map Cπ:C(X)→C(X/σ) between the cone spaces. A simplicial distribution on the cone of the collapsed measurement space can be extended via Cπ to give a simplicial distribution on the cone of the original measurement space. We denote this map by

$${\left(C\pi \right)}^{*}:\mathrm{sDist}(C\left(X/\sigma \right))\to \mathrm{sDist}(C(X)).$$

In Theorem 3, we show that for a simplicial distribution p∈sDist(C(X/σ)) and its image $q={\left(C\pi \right)}^{*}\left(p\right)$, the following holds:

1. *p* is a vertex if and only if *q* is a vertex.
2. *p* is contextual if and only if *q* is contextual.
3. *p* is strongly contextual if and only if *q* is strongly contextual.
4. *p* is a deterministic distribution if and only if *q* is a deterministic distribution.

In particular, parts (1) and (2) imply that contextual vertices map to contextual vertices under the collapsing map. This method is very powerful in detecting vertices. Let $n_{X}$ denote the number of generators of the fundamental group of the graph *X*. Then, the number of contextual vertices in sDist(C(X)) is lower bounded by $\left(2^{n_{X}}-1\right)2^{|X_{0}|-1}$, where $|X_{0}|$ denotes the number of vertices of the graph (Theorem 4). In addition, we use this method to derive new Bell inequalities from known ones. For example, the Froissart inequalities [13] of the scenario given by the cone of the bipartite graph $K_{3,3}$ produce new Bell inequalities for the cone of the graph obtained by collapsing one of the edges (Section 5.1).

Finally, we explore a new algebraic feature of simplicial distributions first introduced in [14]. The set of simplicial distributions sDist(X) has a monoid structure. Together with its polytope structure, this gives a convex monoid. The restriction of the monoid structure to deterministic distributions gives a group structure, and this group acts on simplicial distributions. Using this action, we can generate more vertices from those obtained from the collapsing technique. Our other contributions are as follows: (1) For a two-dimensional measurement space *X*, we show that sDist(X) is a convex polytope (Proposition 4) and provide the *H*-description (Corollary 2). (2) We describe the monoid structure on sDist(X) (Section 2.4) and describe the action of the set of deterministic distributions on Bell inequalities and contextual vertices (Example 3). (3) The one-cycle scenario obtained as the cone of a circle (Figure 17) is a new scenario that cannot be realized in the conventional nonsignaling picture. More generally, we describe the polytope of simplicial distributions on the cone of the wedge $\vee_{i=1}^{n}C_{1}$ of 1-circles (Proposition 11).

## 2. Simplicial Distributions

The theory of simplicial distributions is introduced in [7]. A simplicial distribution is defined for a space of measurements and outcomes. In this formalism, spaces are represented by combinatorial objects known as simplicial sets. More formally, a *simplicial set X* consists of a sequence of sets $X_{n}$ for n≥0 and the simplicial structure maps:

- Face maps $d_{i}:X_{n}\to X_{n-1}$ for 0≤i≤n.
- Degeneracy maps $s_{j}:X_{n}\to X_{n+1}$ for 0≤j≤n.

These maps are subject to simplicial relations (see, e.g., [15]). An *n*-simplex is called *degenerate* if it lies in the image of a degeneracy map, otherwise it is called *nondegenerate*. Geometrically, only the nondegenerate simplices are relevant. Among the nondegenerate simplices, there are ones that are not a face of another nondegenerate simplex. Those simplices we will refer to as *generating simplices*. Throughout the paper, when we refer to an edge (1-simplex) or a triangle (2-simplex) of a simplicial set, we mean a nondegenerate one.

In this paper, we will focus on spaces obtained by gluing triangles.

**Example** **1.** *The triangle, denoted by $\Delta^{2}$, is the simplicial set with simplices*

$${\left(\Delta^{2}\right)}_{n}=\{\sigma^{a_{0}\cdots a_{n}}:\;0\leq a_{0}\leq \cdots a_{n}\leq 2,\;a_{i}\in Z\}.$$

*The i-th face map deletes the i-th index: $d_{i}\left(\sigma^{a_{0}\cdots a_{n}}\right)=\sigma^{a_{0}\cdots a_{i-1}a_{i+1}\cdots a_{n}}$, and the j-th degeneracy map copies the j-th index: $s_{j}\left(\sigma^{a_{0}\cdots a_{n}}\right)=\sigma^{a_{0}\cdots a_{j}a_{j}\cdots a_{n}}$. The simplex $\sigma^{012}$ is the generating simplex. Any other simplex can be obtained by applying a sequence of face and degeneracy maps. In general, we can define $\Delta^{d}$ consisting of n-simplices of the form $\sigma^{a_{0}\cdots a_{n}}$ where $0\leq a_{0}\leq \cdots \leq a_{n}\leq d$. This simplicial set represents the topological d-simplex. Of particular interest are $\Delta^{0}$ and $\Delta^{1}$, representing a point and an edge, respectively.*

The gluing operation can be specified by introducing relations between the generating simplices. The simplest example is obtained by gluing two triangles along a face.

**Example** **2.** *The diamond space D is defined as follows:*

- *Generating simplices: $\sigma_{A}^{012}$ and $\sigma_{B}^{012}$.*
- *Identifying relation: $d_{1}\sigma_{A}^{012}=d_{1}\sigma_{B}^{012}$.*

*We can define other versions by changing the faces. We will write $D_{ij}$ for the diamond whose identifying relation is $d_{i}\sigma_{A}^{012}=d_{j}\sigma_{B}^{012}$.*

Next, we introduce the notion of maps between simplicial sets. A *map f:X→Y of simplicial sets* consists of a sequence $f_{n}:X_{n}\to Y_{n}$ of functions that respect the face and the degeneracy maps. Given a simplex $\sigma \in X_{n}$, we will write $f_{\sigma }$ for $f_{n}\left(\sigma \right)\in Y_{n}$. With this notation, the compatibility conditions are given by

$$d_{i}f_{\sigma }=f_{d_{i}\sigma }\;\;\;\mathrm{and}\;\;\;s_{j}f_{\sigma }=f_{s_{j}\sigma }.$$

A simplicial set map $f:\Delta^{2}\to Y$ is determined by the image of the generating simplex, that is, by an arbitrary 2-simplex $f_{\sigma^{012}}\in Y_{2}$. Therefore, these maps are in bijective correspondence with the elements of $Y_{2}$. In the case of the diamond spacem a simplicial set map $f:D_{ij}\to Y$ is determined by $f_{\sigma_{A}^{012}}$ and $f_{\sigma_{B}^{012}}$, satisfying

$$d_{i}f_{\sigma_{A}^{012}}=f_{d_{i}\sigma_{A}^{012}}=f_{d_{j}\sigma_{B}^{012}}=d_{j}f_{\sigma_{B}^{012}}.$$

Given a simplicial set *Y*, we will construct another simplicial set that represents the space of distributions on *Y*. For this, we need the distribution monad $D_{R}$ defined for a commutative semiring *R* [16]. Throughout the paper, we will take *R* to be $R_{\geq 0}$. A distribution on a set *U* is defined to be a function p:U→R of finite support, such that $\sum_{u\in U}p\left(u\right)=1$. The delta distribution at u∈U is defined by

$$\delta^{u}\left(u^{\prime }\right)=\left\{\begin{array}{cc} 1 & u^{\prime }=u\\ 0 & \mathrm{otherwise}. \end{array}\right.$$

Any distribution can be expressed as a sum of delta distributions: $p=\sum_{u\in U}p\left(u\right)\delta^{u}$. For a function f:U→V, we will write $D_{R}f$ for the function $D_{R}\left(U\right)\to D_{R}\left(V\right)$, defined by

$$D_{R}f\left(p\right)\left(v\right)=\sum_{u\in f^{-1}\left(v\right)}p\left(u\right).$$

The space of distributions on *Y* is represented by the simplicial set $D_{R}\left(Y\right)$, whose *n*-simplices are given by $D_{R}\left(Y_{n}\right)$. The face and the degeneracy maps are given by $D_{R}d_{i}$ and $D_{R}s_{j}$. There is a canonical simplicial set map $\delta :Y\to D_{R}\left(Y\right)$ defined by sending a simplex σ to the delta distribution $\delta^{\sigma }$.

**Definition** **1.** *A simplicialscenario consists of a pair (X,Y) of simplicial sets where X represents the space of measurements and Y represents the space of outcomes. A simplicial distribution on (X,Y) is a simplicial set map $p:X\to D_{R}\left(Y\right)$. A simplicial set map of the form s:X→Y is called an outcome assignment. The associated distribution $\delta^{s}:X\to D_{R}\left(Y\right)$ defined to be the composite δ∘s is called a deterministic distribution.*

*We will write sDist(X,Y) and dDist(X,Y) for the set of simplicial and deterministic distributions.*

There is a canonical map

$$\Theta :D_{R}\left(\mathrm{dDist}(X,Y)\right)\to \mathrm{sDist}(X,Y)$$

defined by sending $d=\sum_{s}d\left(s\right)\delta^{s}$ to the simplicial distribution Θ(p) defined by

$$\Theta {\left(p\right)}_{\sigma }=\sum_{s}d\left(s\right)\delta^{s_{\sigma }}.$$

**Definition** **2.** *A simplicial distribution $p:X\to D_{R}Y$ is called noncontextual if p is in the image of *Θ*. Otherwise, it is called contextual.*

There is a stronger version of contextuality whose definition relies on the notion of support. The *support* of a simplicial distribution $p:X\to D_{R}Y$ is defined by

(1) $$\mathrm{supp}(p)=\{s:X\to Y\;:\;p_{\sigma }\left(s_{\sigma }\right)>0\;\forall \sigma \in X_{n},\;n\geq 0\}.$$

**Definition** **3.** *A simplicial distribution p on (X,Y) is called strongly contextual if its support supp(p) is empty.*

### 2.1. Two-Dimensional Distributions with Binary Outcomes

Throughout the paper, we will work concretely with binary outcome measurements in $Z_{2}$. In effect, this means that our outcome space will be the *nerve space* of $Z_{2}$. This simplicial set is denoted by ${NZ}_{2}$ and is defined as follows:

- The set of *n*-simplices is $Z_{2}^{n}$;
- The face maps are given by
  $$d_{i}\left(a_{1},\cdots ,a_{n}\right)=\left\{\begin{array}{cc} \left(a_{2},\cdots ,a_{n}\right) & i=0\\ \left(a_{1},\cdots ,a_{i}+a_{i+1},\cdots ,a_{n}\right) & 0<i<n\\ \left(a_{1},\cdots ,a_{n-1}\right) & i=n \end{array}\right.$$
  and the degeneracy maps are given by
  $$s_{j}\left(a_{1},\cdots ,a_{n}\right)=\left(a_{1},\cdots ,a_{j},0,a_{j+1},\cdots ,a_{n}\right).$$

Our measurement spaces will be obtained by gluing triangles. A simplicial set is *d-dimensional* if all its nondegenerate simplices are in dimension n≤d. In this paper, we will restrict ourselves to simplicial scenarios of the form $\left(X,{NZ}_{2}\right)$, where *X* is two-dimensional. We will study simplicial distributions on such scenarios. For simplicity of notation, we will write sDist(X), omitting the outcome space when it is fixed to ${NZ}_{2}$, and denote the simplicial scenario only by the measurement space *X*.

Let us look more closely to simplicial distributions on the triangle. Consider a triangle $\Delta^{2}$ with the generating 2-simplex $\sigma =\sigma^{012}$. A simplicial distribution is given by a simplicial set map:

$$p:\Delta^{2}\to D_{R}\left({NZ}_{2}\right)$$

which is determined by the distribution $p_{\sigma }$ on $Z_{2}^{2}$. We will write $p_{\sigma }^{ab}$ for the probability of obtaining the outcome $\left(a,b\right)\in Z_{2}^{2}$ when we measure σ. The three edges bounding σ are given by the face maps as follows: $\sigma^{01}=d_{2}\sigma^{012}$, $\sigma^{02}=d_{1}\sigma^{012}$, $\sigma^{12}=d_{0}\sigma^{012}$. For simplicity of notation, we will write $x=\sigma^{01}$, $y=\sigma^{12}$ and $z=\sigma^{02}$. The corresponding marginal distribution $p_{x}:Z_{2}\to R_{\geq 0}$ at edge *x* can be identified with $\left(p_{x}^{0},p_{x}^{1}\right)$. Since $p_{x}^{0}+p_{x}^{1}=1$, it suffices just to keep $p_{x}^{0}$. Similarly for edges *y* and *z*. Compatibility with face maps requires that

$$\begin{array}{ccc} p_{x}^{0} & = & p_{\sigma }^{00}+p_{\sigma }^{01},\\ p_{y}^{0} & = & p_{\sigma }^{00}+p_{\sigma }^{10},\\ p_{z}^{0} & = & p_{\sigma }^{00}+p_{\sigma }^{11}. \end{array}$$

Since $p_{\sigma }^{ab}$ is also normalized, it can be expressed by three parameters. Without loss of generality, we can take these three parameters to be the marginal distributions corresponding to the edges on the boundary. Conversely, given the marginals on the edges, we have that

(2) $$\begin{array}{ccc} p_{\sigma }^{ab} & = & \frac{1}{2}\left(p_{x}^{a}+p_{y}^{b}-p_{z}^{a+b+1}\right). \end{array}$$

Therefore, a simplicial distribution on the triangle is determined by its restriction to the boundary. This observation generalizes to every two-dimensional simplicial set. As we will observe in Proposition 4, for such measurement spaces, restriction of a simplicial distribution to the one-dimensional simplicial subset consisting of all the edges determines the distribution. Alternatively, we can use the expectation coordinates instead of the probability coordinates. For an edge $\tau \in X_{1}$, let us define its expectation value by

(3) $$\begin{array}{c} \bar{\tau }=p_{\tau }^{0}-p_{\tau }^{1}. \end{array}$$

Using this, we can rewrite $p_{\sigma }^{ab}$, which takes the form

(4) $$\begin{array}{c} p_{\sigma }^{ab}=\frac{1}{4}\left(1+{\left(-1\right)}^{a}\bar{x}+{\left(-1\right)}^{b}\bar{y}+{\left(-1\right)}^{a+b}\bar{z}\right). \end{array}$$

Next we describe noncontextual distributions on $\Delta^{2}$. Let us start with outcome assignments. An outcome assignment $s:\Delta^{2}\to {NZ}_{2}$ is determined by a pair of bits $s_{\sigma }\in Z_{2}^{2}$. The corresponding deterministic distribution is $\delta^{s}$. For simplicity of notation, we will write $\delta^{ab}$ for the deterministic distribution corresponding to the outcome assignment $s_{\sigma }=\left(a,b\right)$.

**Proposition** **1.** *Every simplicial distribution on $\Delta^{2}$ is noncontextual.*

**Proof.**  Given a simplicial distribution $p:\Delta^{2}\to D\left({NZ}_{2}\right)$ described by ${\left\{p_{\sigma }^{ab}\right\}}_{a,b\in Z_{2}}$. Then the classical distribution

$$d=\sum_{a,b}d\left(ab\right)\delta^{ab}\;\;\mathrm{with}\;d\left(ab\right)=p_{\sigma }^{ab}$$

satisfies Θ(d)=p. □

In this paper, we are interested in cones of one-dimensional simplicial sets. For instance, the *N*-cycle scenario (Definition 11) is of this form. Given a simplicial set *X*, we will construct a new simplicial set denoted by C(X), which represents the topological construction of adding a new vertex and joining every *n*-simplex of *X* to this vertex to create an (n+1)-simplex. The new vertex is represented by $\Delta^{0}$, the simplicial set representing a point. This simplicial set is defined by

- ${\left(\Delta^{0}\right)}_{n}=\{\sigma^{0\cdots 0}\}$,
- the face and the degeneracy maps are given by deleting and copying; see Example 1.

For notational convenience, we will write $c_{n}$ for the simplex $\sigma^{0\cdots 0}$ in dimension *n*. With this notation, a face map sends $c_{n}\mapsto c_{n-1}$ and a degeneracy map sends $c_{n}\mapsto c_{n+1}$.

**Definition** **4.** *The cone C(X) is the simplicial set given as follows:*

- *${\left(C\left(X\right)\right)}_{n}=\{c_{n}\}⊔X_{n}⊔\left({⊔}_{k+1+l=n}\{c_{k}\}\times X_{l}\right)$.*
- *For $\left(c_{k},\sigma \right)\in \{c_{k}\}\times X_{l}$*
$$d_{i}\left(c_{k},\sigma \right)=\left\{\begin{array}{cc} \left(c_{k-1},\sigma \right) & i\leq k\\ \left(c_{k},d_{i-1-k}\sigma \right) & i>k \end{array}\right.$$
*and*
$$s_{j}\left(c_{k},\sigma \right)=\left\{\begin{array}{cc} \left(c_{k+1},\sigma \right) & j\leq k\\ \left(c_{k},s_{j-1-k}\sigma \right) & j>k. \end{array}\right.$$
*Otherwise, the face and the degeneracy maps on the $\left\{c_{n}\right\}$ and $X_{n}$ factors act the same as in $\Delta^{0}$ and X.*

This construction is a special case of the join construction Z*X defined for a pair of simplicial sets (Chapter 17.1 in [17]). In the cone construction, $Z=\Delta^{0}$. We will use the cone construction to obtain two-dimensional measurement spaces.

**Remark** **1.** *For n≥1, the nondegenerate n-simplices of C(X) are of the form $\left(c_{0},\sigma \right)$ where σ is a nondegenerate (n−1)-simplex of X. We will usually write $c=c_{0}$.*

### 2.2. Gluing and Extending Distributions

Fundamental tools in the study of simplicial distributions are the extension and the gluing lemmas. They will be crucial for the proof of Fine’s theorem for the *N*-cycle and the flower scenarios in Section 4. Given a simplicial set map f:Z→X, we will write

$$f^{*}:\mathrm{sDist}(X)\to \mathrm{sDist}(Z)$$

for the map that sends a simplicial distribution *p* on *X* to the simplicial distribution defined by the composition $f^{*}p:Z\overset{f}{\to }X\overset{p}{\to }D_{R}\left({NZ}_{2}\right)$. Similarly, there is a map between the deterministic distributions, which is also denoted by $f^{*}:\mathrm{dDist}(X)\to \mathrm{dDist}(Z)$. In this case, a deterministic distribution $\delta^{s}$ is sent to $\delta^{s\circ f}$. There is a commutative diagram:

(5) $$\begin{array}{ccc} D_{R}\mathrm{dDist}(\left(X\right))\; & \overset{\Theta }{\;\to \;} & \mathrm{sDist}(\left(X\right))\\ ↓D_{R}f^{*} & \; & ↓f^{*}\\ D_{R}\left(\mathrm{dDist}(Z)\right)\; & \overset{\Theta }{\;\to \;} & \mathrm{sDist}(Z) \end{array}$$

**Proposition** **2.** If q∈sDist(X) is noncontextual then $p=f^{*}\left(q\right)$ is also noncontextual.

**Proof.**  Let $d\in D_{R}\left(\mathrm{dDist}\left(X\right)\right)$, such that Θ(d)=q. Then, $e=D_{R}f^{*}\left(d\right)$ satisfies Θ(e)=p by the commutativity of Diagram (5). □

Let *A* be a simplicial subset of *X* and let us write i:A→X for the inclusion map. This means that each $A_{n}$ is a subset of $X_{n}$ and the simplicial structure of *A* is compatible with that of *X*. Given p∈sDist(X), we will write ${p|}_{A}$ for the distribution $i^{*}p$. For a deterministic distribution $\delta^{s}$ on *X*, the distribution $i^{*}\delta^{s}$ will be denoted by $\delta^{s}{|}_{A}$. Note that $\delta^{s}{|}_{A}=\delta^{{s|}_{A}}$, where ${s|}_{A}$ stands for the composition ${s|}_{A}:A\overset{i}{\to }X\overset{s}{\to }{NZ}_{2}$.

An important special case of Proposition 2 is the following result, which we will need later in the paper.

**Corollary** **1.** *Let A be a simplicial subset of X. If q∈sDist(X) is noncontextual, then ${q|}_{A}$ is also noncontextual.*

Another important result is the following Gluing Lemma. Using this result, one can reduce the study of distributions on a measurement space to its smaller constituents in some cases.

**Lemma** **1.** *Suppose that X=A∪B with $A\cap B=\Delta^{n}$ for some n≥0. Then, p∈sDist(X) is noncontextual if and only if both ${p|}_{A}\in \mathrm{sDist}(A)$ and ${p|}_{B}\in \mathrm{sDist}(B)$ are noncontextual.*

**Proof.**  See Corollary 4.6 in [7]. □

### 2.3. Polytope of Simplicial Distributions

Recall that the triangle $\Delta^{2}$ has a single generating simplex *σ*. The boundary ∂σ consists of three nondegenerate 1-simplices denoted by x,y,z. Using Equation (2), the polytope of simplicial distributions $\mathrm{sDist}(\Delta^{2})$ can be described as the space consisting of triples $\left(p_{x}^{0},p_{y}^{0},p_{z}^{0}\right)\in R^{3}$ satisfying

(6) $$\begin{array}{cc} p_{x}^{0}+p_{y}^{0}+p_{z}^{0} & \geq 1\\ p_{x}^{0}-p_{y}^{0}-p_{z}^{0} & \geq -1\\ -p_{x}^{0}+p_{y}^{0}-p_{z}^{0} & \geq -1\\ -p_{x}^{0}-p_{y}^{0}+p_{z}^{0} & \geq -1. \end{array}$$

This set of inequalities is an example of *N*-circle inequalities introduced in Definition 9 (N=3). They imply that $\mathrm{sDist}(\Delta^{2})$ is a tetrahedron in $R^{3}$. Proposition 1 can be used to observe that its vertices are given by (a,b,c), where a,b,c∈{0,1} and c+1=a+bmod2.

In general, we will show that sDist(X) is described as the intersection of finitely many half-space inequalities corresponding to the non-negativity of the parameters $p_{\sigma }^{ab}$. Such a description of a polytope is called the *H*-representation. Our goal is to characterize the geometric structure of sDist(X) including the vertices (extreme distributions) and the Bell inequalities bounding the noncontextual distributions.

**Definition** **5.** *A 1-simplex τ of X is called a deterministic edge (with respect to p) if $p_{\tau }$ is a deterministic distribution on $Z_{2}$.*

**Proposition** **3.** *If two of the edges of a triangle are deterministic, then the third edge is also deterministic.*

**Proof.**  Assume that $p_{x}^{0}=1$ and $p_{y}^{0}=1$; the other cases follow similarly. Then, the last inequality in Equation (6) implies that $p_{z}^{0}=1$. □

Next, we recall some basic facts from polytope theory [11,18]. In the *H*-representation, a (convex) polytope is specified by a set of inequalities:

$$P\left(A,b\right)=\{x\in R^{d}:\;Ax\geq b\}$$

where *A* is a m×d matrix and *b* is a column vector of size *m*. We will assume that $P\subset R^{d}$ is full-dimensional, that is, the dimension of the polytope is given by *d*.

**Lemma** **2.** *Let X be a *2*-dimensional simplicial set with a single generating *2*-simplex σ whose boundary ∂σ consists of the *1*-simplices x,y,z all of them are non-degenerate simplices. Consider the injective map*

$$f_{\sigma }:\mathrm{sDist}(X)\to {\left[0,1\right]}^{\left|\partial \sigma \right|}$$

*that sends p to the tuple ${\left(p_{\tau }^{0}\right)}_{\tau \in \partial \sigma }$. Then, the image of f is a polytope of dimension |∂σ|.*

**Proof.**  Let *P* denote the image of *f*. First, consider the case where |∂σ|=3. *P* is defined by the set of inequalities in Equation (6). This is a tetrahedron in $R^{3}$ with vertices $\left(\delta^{a},\delta^{b},\delta^{c}\right)$, where a,b,c∈{0,1} and c=a+bmod2. Therefore, the dimension of *P* is 3. Next, consider |∂σ|=2. We can assume that *x* and *y* are identified. Then, the polytope is obtained by intersecting the tetrahedron by the hyperplane $p_{x}^{0}=p_{y}^{0}$. This gives a two-dimensional polytope. Finally, if |∂σ|=1, then all the edges are identified. The polytope is obtained by intersecting the previous one with $p_{y}^{0}=p_{z}^{0}$ producing a polytope of dimension 1. □

A polytope $P\left(A,b\right)\subset R^{d}$ is called full-dimensional if the dimension of the polytope is *d*. For a simplicial set *X*, we will write $X_{n}^{\circ }$ for the set of nondegenerate simplices. Let $X^{\left(n\right)}$ denote the simplicial subset of *X* generated by $X_{n}^{\circ }$. For example, $X^{\left(1\right)}$ is generated by non-degenerate 1-simplices together with the face relations coming from *X*.

**Proposition** **4.** *Let X be a simplicial set generated by the *2*-simplices $\sigma_{1},\cdots ,\sigma_{k}$, such that each $\partial \sigma_{i}$ does not contain nondegenerate edges. The map*

(7) $$f:\mathrm{sDist}(X)\to \left(\mathrm{sDist}X^{\left(1\right)}\right)={\left[0,1\right]}^{|X_{1}^{\circ }|}$$

*that sends p to the tuple ${\left(p_{\tau }^{0}\right)}_{\tau \in X_{1}^{\circ }}$ is a convex injective map. Moreover, $P_{X}\subset R^{|X_{1}^{\circ }|}$ is a full-dimensional convex polytope.*

**Proof.**  This follows from Lemma 2: For each $\sigma_{i}$, the restriction of *f* to the simplicial set $X_{i}$ generated by $\sigma_{i}$ gives a map:

$$f_{\sigma }:\mathrm{sDist}(X_{i})\to {\left[0,1\right]}^{|\partial \sigma_{i}|}.$$

Thus, $P_{X_{i}}$ is a full-dimensional polytope. Consider the projection map $R^{|X_{1}^{\circ }|}\to R^{|\partial \sigma_{i}|}$ onto the coordinates of the boundary. Combining these projections, we can obtain a linear embedding $i:R^{|X_{1}^{\circ }|}\to \prod_{i=1}^{k}R^{|\partial \sigma_{i}|}$. Then, $P_{X}$ is given by the intersection of the image of *i* and the product of the polytopes $\prod_{i=1}^{k}P_{X_{i}}$. This intersection remains to be full-dimensional in the linear subspace. □

In practice, this result implies that a simplicial distribution on a two-dimensional simplicial set is determined by its restriction to the edges. This description of *p* will be referred to as the edge coordinates. With this result at hand, it is straightforward to give the *H*-description of $P_{X}$.

**Corollary** **2.** *Let $d_{X}=X_{1}^{\circ }$ and $m_{X}=\left|X_{2}^{\circ }\times Z_{2}^{2}\right|$. We define an $m_{X}\times d_{X}$ matrix:*

$$A_{(\sigma ;ab),\tau }=\left\{\begin{array}{cc} {\left(-1\right)}^{a} & \tau =x\\ {\left(-1\right)}^{b} & \tau =y\\ {\left(-1\right)}^{a+b+1} & \tau =z\\ 0 & \mathrm{otherwise}, \end{array}\right.$$

*and a column vector b of size $m_{X}$:*

$$b_{\left(\sigma ,ab\right)}=\frac{\left({\left(-1\right)}^{a}+{\left(-1\right)}^{b}-{\left(-1\right)}^{a+b}\right)-1}{2}.$$

*Then, $P_{X}$ is described as P(A,b).*

We adopt a notation where if Ƶ⊆{1,⋯,m}, then A[Ƶ] is the matrix obtained by keeping only those rows indexed by *Ƶ* and discarding the rest, and similarly for b[Ƶ]. Let i∈{1,⋯,m} index a single inequality and x∈P; then, we call an inequality *i* at *x tight* if the inequality is satisfied with equality, i.e., $A_{i}x=b_{i}$. For a point x∈P, we write ${Ƶ}_{x}$ for the set of tight inequalities at *x*.

**Definition** **6.** *The rank rank(p) of a simplicial distribution p∈sDist(X) is defined to be the rank of the matrix $A[{Ƶ}_{p}]$.*

**Corollary** **3.** *A simplicial distribution p∈sDist(X) is a vertex if and only if $rank(p)=|X_{1}^{\circ }|$.*

**Proof.**  For a full-dimensional polytope $P\subset R^{d}$, a point v∈P is a vertex if and only if it is the unique solution to *d* tight inequalities. More explicitly, if Ƶ⊂{1,⋯,m} indexes *d* inequalities such that A[Ƶ] has full rank, then a vertex is given by

$$v=A{\left[Ƶ\right]}^{-1}b.$$

This basic fact applied to $P_{X}$, where $d=|X_{1}^{\circ }|$, combined with Proposition 4, gives the result. □

### 2.4. Monoid Structure on Simplicial Distributions

An additional algebraic feature that comes for free in the theory of simplicial distributions is the monoid structure on sDist(X,Y) when *Y* is a simplicial set, which also has the structure of a group. Such a group-like simplicial set is called a simplicial group.

Our outcome space $NZ_{2}$ has this additional algebraic feature, which comes from the following simplicial set map:

$$\cdot :NZ_{2}\times NZ_{2}\to NZ_{2}$$

defined by

(8) $$\left(a_{1},\cdots ,a_{n}\right)\cdot \left(b_{1},\cdots ,b_{n}\right)=\left(a_{1}+b_{1},\cdots ,a_{n}+b_{n}\right).$$

It is straightforward to verify that this assignment respects the face and the degeneracy maps. This product gives the set dDist(X) of deterministic distributions the structure of a group. Given two such distributions $\delta^{s}$ and $\delta^{r}$, their product is given by $\delta^{s\cdot r}$, where

$$s\cdot r:X\overset{\left(s,r\right)}{\to }NZ_{2}\times NZ_{2}\overset{\cdot }{\to }NZ_{2}.$$

We will write $\delta^{s}\cdot \delta^{r}$ to denote this product of deterministic distributions.

**Lemma** **3.**

*1.*  *The product on $\mathrm{dDist}(\Delta^{1})$ is given by*
$$\delta^{a}\cdot \delta^{b}=\delta^{a+b}.$$
*2.*  *The product on $\mathrm{dDist}(\Delta^{2})$ is given by*
$$\delta^{ab}\cdot \delta^{cd}=\delta^{\left(a+c)(b+d\right)}.$$

**Proof.**  Let $\tau =\sigma^{01}$ denote the generating simplex of $\Delta^{1}$. Consider two deterministic distributions $\delta^{s}$ and $\delta^{r}$, such that $s_{\tau }=a$ and $r_{\tau }=b$. The product s·r is determined by its value at *τ*. Using Equation (8), we have

$${\left(s\cdot r\right)}_{\tau }=a\cdot b=a+b.$$

For $\Delta^{2}$, we will consider the generating simplex $\sigma =\sigma^{012}$. By a similar argument applied to $s_{\sigma }=\left(a,b\right)$ and r=(c,d), we observe that

$${\left(s\cdot r\right)}_{\sigma }=\left(a,b\right)\cdot \left(c,d\right)=\left(a+c,b+d\right).$$

□

Lemma 3 can be used to describe the product on dDist(X) when *X* is two-dimensional. This product can be extended to D(dDist). Given d,e∈D(dDist(X)), we define

$$\left(d\cdot e\right)\left(s\right)=\sum_{r\cdot t=s}d\left(r\right)d\left(t\right)$$

where the summation runs over $\left(\delta^{r},\delta^{t}\right)\in {\left(\mathrm{dDist}\left(X\right)\right)}^{2}$ satisfying r·t=s. With this product, D(dDist(X)) is a monoid. Next, we turn to the monoid structure on sDist(X). Given two simplicial distributions p,q on *X*, the product p·q is defined by

(9) $${\left(p\cdot q\right)}_{\sigma }^{a}=\sum_{b+c=a}p_{\sigma }^{b}q_{\sigma }^{c}$$

where the summation runs over $\left(b,c\right)\in {\left(Z_{2}^{n}\right)}^{2}$, satisfying b+c=a. This formula works for an n-simplex *σ*. For us, the main interest is the cases n=1,2.

**Lemma** **4.** *Let X be a simplicial set and p,q∈sDist(X).*

*1.*  *For $\tau \in X_{1}$, we have*
$${\left(p\cdot q\right)}_{\tau }^{0}=p_{\tau }^{0}\cdot q_{\tau }^{0}+p_{\tau }^{1}\cdot q_{\tau }^{1},\;\;\;\;{\left(p\cdot q\right)}_{\tau }^{1}=p_{\tau }^{0}\cdot q_{\tau }^{1}+p_{\tau }^{1}\cdot q_{\tau }^{0}$$
*2.*  *For $\sigma \in X_{2}$, we have*
$$\begin{array}{ccc} {\left(p\cdot q\right)}_{\sigma }^{00} & = & p_{\sigma }^{00}\cdot q_{\sigma }^{00}+p_{\sigma }^{01}\cdot q_{\sigma }^{01}+p_{\sigma }^{10}\cdot q_{\sigma }^{10}+p_{\sigma }^{11}\cdot q_{\sigma }^{11},\\ {\left(p\cdot q\right)}_{\sigma }^{01} & = & p_{\sigma }^{00}\cdot q_{\sigma }^{01}+p_{\sigma }^{01}\cdot q_{\sigma }^{00}+p_{\sigma }^{10}\cdot q_{\sigma }^{11}+p_{\sigma }^{11}\cdot q_{\sigma }^{10},\\ {\left(p\cdot q\right)}_{\sigma }^{10} & = & p_{\sigma }^{00}\cdot q_{\sigma }^{10}+p_{\sigma }^{01}\cdot q_{\sigma }^{11}+p_{\sigma }^{10}\cdot q_{\sigma }^{00}+p_{\sigma }^{11}\cdot q_{\sigma }^{01},\\ {\left(p\cdot q\right)}_{\sigma }^{11} & = & p_{\sigma }^{00}\cdot q_{\sigma }^{11}+p_{\sigma }^{01}\cdot q_{\sigma }^{10}+p_{\sigma }^{10}\cdot q_{\sigma }^{01}+p_{\sigma }^{11}\cdot q_{\sigma }^{00} \end{array}$$

**Proof.**  Follows directly from Equation (9). □

Moreover, the map Θ:D(dDist(X))→sDist(X) is a homomorphism of monoids. For more on the monoid structure and its interaction with convexity, see [14]. We will use the action of the group dDist(X) on the monoid sDist(X) that comes from the product in Equation (9). Explicitly, for $\sigma \in X_{2}$ and $\tau \in X_{1}$, this action is described as follows:

(10) $${\left(\delta^{a}\cdot q\right)}_{\tau }^{c}=q_{\tau }^{c+a},\;\;\;\;{\left(\delta^{ab}\cdot q\right)}_{\sigma }^{cd}=q_{\sigma }^{\left(c+a)(d+b\right)}.$$

Note that this action maps vertices of sDist(X) to vertices.

**Proposition** **5.**

*1.*  *For two noncontextual simplicial distributions p and q in sDist(X), the product p·q is a noncontextual distribution.*
*2.*  *A simplicial distribution p∈sDist(X) is noncontextual if and only if $\delta^{s}\cdot p$ is noncontextual.*
*3.*  *A simplicial distribution p∈sDist(X) is a vertex if and only if $\delta^{\varphi }\cdot p$ is a vertex.*

**Proof.**  Part 1 follows from the fact that the map Θ:D(dDist(X))→sDist(X) is a homomorphism of monoids (Lemma 5.1 in [14]). Part 2 follows from Part 1. □

Part (2) of this proposition implies that the action of dDist(X) on sDist(X) maps a (non)contextual vertex to a (non)contextual vertex. We describe the action in the case of the well-known CHSH scenario in Example 3 below.

The following simplicial distributions on $\Delta^{2}=C(\Delta^{1})$ will play a distinguished role in later sections when we study two-dimensional scenarios more closely:

(11) $$p_{+}^{ab}=\left\{\begin{array}{cc} 1/2 & b=0\\ 0 & \mathrm{otherwise}. \end{array}\right.\;\;\;\;p_{-}^{ab}=\left\{\begin{array}{cc} 0 & b=0\\ 1/2 & \mathrm{otherwise}. \end{array}\right.$$

**Figure 2:**
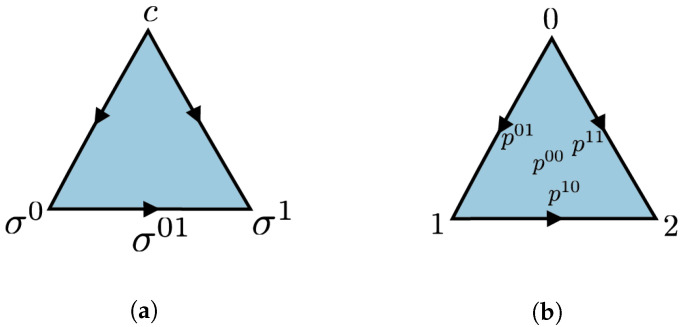
(**a**) A triangle can be considered as the cone of an edge. The generating 2-simplex is given by $\left(c,\sigma^{01}\right)$ whose faces are $\left(c,\sigma^{0}\right)$, $\left(c,\sigma^{1}\right)$ and $\sigma^{01}$, where $c=c_{0}$. (**b**) A simplicial distribution on the triangle.

We follow the convention in Figure 2b.

**Definition** **7.** *Let X be a one-dimensional simplicial set. We will write $G_{\pm }\left(CX\right)$ for the subset of simplicial distributions p∈sDist(CX) satisfying ${p|}_{\left(c,\tau \right)}=p_{\pm }$ for every $\tau \in X_{1}^{\circ }$.*

Next, we show that this set is a group. We will denote the distribution in $G_{\pm }\left(CX\right)$ with ${p|}_{\left(c,\tau \right)}=p_{+}$ for every $\tau \in X_{1}^{\circ }$ by $e_{+}$.

**Proposition** **6.** *$G_{\pm }\left(CX\right)$ is an abelian group with $e_{+}$ as the identity. In addition, every element has order 2, that is,*

$$G_{\pm }\left(CX\right)≅Z_{2}^{X_{1}^{\circ }}.$$

**Proof.**  By Part 2 of Lemma 4, we have

$$p_{+}\cdot p_{+}=p_{+}\;\;,\;\;p_{+}\cdot p_{-}=p_{-}\;\;,\;\;p_{-}\cdot p_{-}=p_{+}\;\;,\;\;$$

Therefore, the statement holds for $X=\Delta^{1}$, that is, we have

$$G_{\pm }\left(C\left(\Delta^{1}\right)\right)≅Z_{2}.$$

Now for arbitrary *X* and $p,q\in G_{\pm }\left(CX\right)$, the product is computed triangle-wise, i.e., ${\left(p\cdot q\right)}_{\sigma }=p_{\sigma }\cdot q_{\sigma }$. Therefore, the statement easily generalizes. □

**Figure 3:**
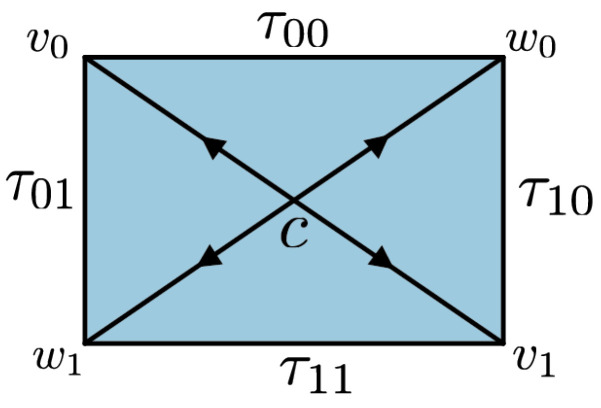
The CHSH scenario as the cone of the circle consisting of $\tau_{00},\tau_{01},\tau_{10},\tau_{11}$ (Definition 10).

**Example** **3.** *The CHSH scenario consists of four triangles organized into a disk with vertices $v_{0},v_{1},w_{0},w_{1}$, and c. For each pair $\left(v_{i},w_{j}\right)$, there is an edge, which we denote by $\tau_{ij}$. This constitutes the boundary of the disk. There are four nondegenerate triangles $\sigma_{ij}=\left(c,\tau_{ij}\right)$, as depicted in Figure 3. The interior edges $\left(c,v_{i}\right)$ and $\left(c,w_{j}\right)$ will be denoted by $x_{i}$ and $y_{j}$, respectively. This scenario is a particular case of the N-cycle scenario in Definition 11. Here, N is the number of edges on the boundary; hence, in this case, N=4. Using the edge coordinates of Proposition 4, a simplicial distribution p on the CHSH scenario can be described by the tuple $\left(p_{x_{0}},p_{\tau_{00}},p_{y_{0}},p_{\tau_{10}},p_{x_{1}},p_{\tau_{11}},p_{y_{1}},p_{\tau_{01}}\right)$. It is well known that p is noncontextual if and only if it satisfies the CHSH inequalities [4]:*

(12) $$\begin{array}{c} 0\leq p_{\tau_{00}}^{0}+p_{\tau_{10}}^{0}+p_{\tau_{11}}^{0}-p_{\tau_{01}}^{0}\leq 2\\ 0\leq p_{\tau_{00}}^{0}+p_{\tau_{10}}^{0}-p_{\tau_{11}}^{0}+p_{\tau_{01}}^{0}\leq 2\\ 0\leq p_{\tau_{00}}^{0}-p_{\tau_{10}}^{0}+p_{\tau_{11}}^{0}+p_{\tau_{01}}^{0}\leq 2\\ 0\leq -p_{\tau_{00}}^{0}+p_{\tau_{10}}^{0}+p_{\tau_{11}}^{0}+p_{\tau_{01}}^{0}\leq 2 \end{array}$$

*Also, the contextual vertices are known. They are given by the Popescu–Rohrlich (PR) boxes [19]: A PR box is a simplicial distribution p, such that $p_{\sigma_{ij}}=p_{\pm }$ for i,j∈{0,1}, with the further restriction that the number of $p_{-}$s is odd.*

*We begin with the action on the PR boxes. By Equation (10), we see that $\delta^{00}$ and $\delta^{10}$ are the only deterministic distributions on the triangles that fix $p_{+}$ and $p_{-}$. From this observation, we conclude that among the 16 deterministic distributions on the CHSH scenario, the ones that fix a given PR box are $\left(\delta_{\sigma_{00}}^{00},\delta_{\sigma_{01}}^{00},\delta_{\sigma_{10}}^{00},\delta_{\sigma_{11}}^{00}\right)$ and $\left(\delta_{\sigma_{00}}^{10},\delta_{\sigma_{01}}^{10},\delta_{\sigma_{10}}^{10},\delta_{\sigma_{11}}^{10}\right)$. Thus, the size of the orbit is 16/2=8, which gives all the PR boxes.*

*To describe the action of $\delta^{s}$ on the Bell inequalities, we need to switch back to the edge coordinates. For notational convenience, we will write $p_{i}$ for the i-th entry of this tuple. Then, the deterministic distribution $\delta^{s}$ is given by $\left(\delta^{a_{0}},\delta^{a_{0}+b_{0}},\delta^{b_{0}},\delta^{a_{1}+b_{0}},\delta^{a_{1}},\delta^{a_{1}+b_{1}},\delta^{b_{1}},\delta^{a_{0}+b_{1}}\right)$. Using the notational convenience introduced above, in these coordinates, the action of $\delta^{s}$ on p is given by*

$${\left(\delta^{s}\cdot p\right)}_{i}={\left(\delta^{s}\right)}_{i}\cdot p_{i}.$$

*Now, substituting these new values to the Bell inequality gives the action. For example, the action of $\left(\delta^{0},\delta^{1},\delta^{1},\delta^{0},\delta^{1},\delta^{1},\delta^{0},\delta^{0}\right)$ on the Bell inequality*

(13) $$p_{\sigma_{00}}^{0}+p_{\sigma_{10}}^{0}+p_{\sigma_{11}}^{0}-p_{\sigma_{01}}^{0}\leq 2$$

*gives $1-p_{\sigma_{00}}^{0}+p_{\sigma_{10}}^{0}+1-p_{\sigma_{11}}^{0}-p_{\sigma_{01}}^{0}\leq 2$, which can be put in a more familiar form*

$$p_{\sigma_{00}}^{0}-p_{\sigma_{10}}^{0}+p_{\sigma_{11}}^{0}+p_{\sigma_{01}}^{0}\geq 0.$$

*We can compute the stabilizer of the Bell inequality in Equation (13). The relevant edge coordinates are $\sigma_{ij}$, which constitute the boundary of the CHSH scenario. The relevant coordinates of $\delta^{s}$ that can change the inequality are $\delta^{a_{i}+b_{j}}$, where i,j∈{0,1}. Then, the stabilizer consists of those deterministic distributions that satisfy $a_{i}+b_{j}=0\;\mathrm{mod}\;2$ for every i,j∈{0,1}. The size of this group is *2*, and therefore, there are 8=16/2 elements in the orbit. This covers all eight of the Bell inequalities.*

See Section 5.1 for more on the action on Bell inequalities.

## 3. Distributions on the Classical *N*-Disk

The classical *N*-disk scenario has the measurement space given by a disk triangulated in a way that results in only noncontextual (or classical) distribution.

**Definition** **8.** *For N≥3, let $D_{N}$ denote the following simplicial set:*

- *Generating *2*-simplices: $\sigma_{1},\cdots ,\sigma_{N-2}$.*
- *Identifying relations:*
$$d_{j_{1}}\left(\sigma_{1}\right)=d_{j_{2}}\left(\sigma_{2}\right),\;d_{j_{2}^{\prime }}\left(\sigma_{2}\right)=d_{j_{3}}\left(\sigma_{3}\right),\;d_{j_{3}^{\prime }}\left(\sigma_{3}\right)=d_{j_{4}}\left(\sigma_{4}\right)\;\cdots \;d_{j_{N-3}^{\prime }}\left(\sigma_{N-3}\right)=d_{j_{N-2}}\left(\sigma_{N-2}\right)$$
  *where $j_{1},j_{2},j_{2}^{\prime },\cdots ,j_{N-3},j_{N-3}^{\prime },j_{N-2}\in \left\{0,1,2\right\}$ and $j_{k}\neq j_{k}^{\prime }$ for 2≤k≤N−3.*

The classical *N*-disk can be constructed by successive gluing. To see this, starting from an initial nondegenerate simplex $\sigma_{1}$, we successively glue simplices along a single edge so that $\sigma_{i+1}$ shares a single common edge with $\sigma_{i}$, terminating with the simplex $\sigma_{N-2}$. The simplices $\sigma_{1}$ and $\sigma_{N-2}$ in any classical *N*-disk will be referred to as the initial and terminal simplices, respectively. In particular, the gluing described by the face relations is such that the boundary of the disk has *N* edges and forms an *N*-circle in the sense of Definition 10. Letting ${\left(\partial D_{N}\right)}_{1}^{\circ }$ be the nondegenerate edges on the boundary of the classical *N*-disk, nondegenerate simplicies in the classical *N*-disk are distinguished by

(14) $$\begin{array}{c} |{\left(\partial D_{N}\right)}_{1}^{\circ }\cap {\left(\sigma_{i}\right)}_{1}^{\circ }|=\left\{\begin{array}{cc} 2 & i=1,N-2\\ 1 & \mathrm{otherwise}. \end{array}\right.\;\;\;\; \end{array}$$

**Figure 4:**
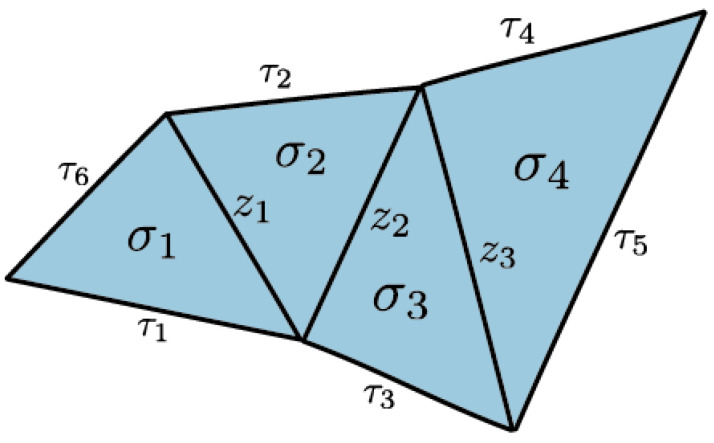
Classical 6-disk with initial simplex $\sigma_{1}$ and terminal simplex $\sigma_{4}$.

Such edges in the classical N-disk are called boundary edges, otherwise we call them interior edges. The classical 3-disk is $\Delta^{2}$, while the diamond space *D* is an example of a classical 4-disk. See Figure 4 for an example of a classical 6-disk.

**Proposition** **7.** *Any simplicial distribution on the classical N-disk scenario is noncontextual.*

**Proof.**  This follows from (Gluing) Lemma 1, since $D_{N}$ is constructed by gluing *N* triangles along a $\Delta^{1}$. At each step, we can apply the Gluing Lemma. □

### 3.1. Fourier–Motzkin Elimination

As is well known, systems of linear equations can be solved using Gaussian elimination. For systems of linear inequalities, there exists a related technique known as Fourier–Motzkin (FM) elimination; see, e.g., [18]. A linear inequality in *d* variables can be written as $a^{T}x\geq b$, where $a,x\in R^{d}$ and b∈R. For *m* such linear inequalities, we have $a_{i}^{T}x\geq b_{i}$ (i=1,⋯,m). Taking each vector $a_{i}^{T}$ to be a row of a matrix *A*, this set of *m* inequalities can be compactly written as Ax≥B, where $A\in R^{m\times d}$ and $B\in R^{m}$. The feasible region defined by Ax≥B (if one exists) forms a polyhedron.

To perform FM elimination of a variable $x_{j}$, let us first index all inequalities where $x_{j}$ appears with positive, negative, or zero coefficients as $I_{j}^{+}$, $I_{j}^{-}$, and $I_{j}^{0}$, respectively. We then solve for $x_{j}$:

$$\begin{array}{ccc} x_{j} & \geq & \frac{B_{i}}{a_{ij}}-\sum_{k\neq j}\frac{a_{ik}}{a_{ij}}x_{k},\;\forall i\in I_{j}^{+},\\ x_{j} & \leq & -\frac{B_{i}}{|a_{ij}|}+\sum_{k\neq j}\frac{a_{ik}}{|a_{ij}|}x_{k},\;\forall i\in I_{j}^{-}. \end{array}$$

Then, for every $\left(i,i^{\prime }\right)\in I_{j}^{+}\times I_{j}^{-}$, we have that such an $x_{j}$ exists so long as

$$\begin{array}{c} \frac{B_{i}}{a_{ij}}-\sum_{k\neq j}\frac{a_{ik}}{a_{ij}}x_{k}\leq x_{j}\leq -\frac{B_{i^{\prime }}}{|a_{i^{\prime }j}|}+\sum_{k\neq j}\frac{a_{i^{\prime }k}}{|a_{i^{\prime }j}|}x_{k}, \end{array}$$

which is equivalent to

(15) $$\begin{array}{c} \frac{B_{i}}{a_{ij}}-\sum_{k\neq j}\frac{a_{ik}}{a_{ij}}x_{k}\leq -\frac{B_{i^{\prime }}}{|a_{i^{\prime }j}|}+\sum_{k\neq j}\frac{a_{i^{\prime }k}}{|a_{i^{\prime }j}|}x_{k}. \end{array}$$

This can be rearranged to give a new set of inequalities in d−1 variables whose solution, should it exist, is the same as the original set of inequalities.

#### Application to the Diamond Scenario

As a warm up, we begin by considering the diamond scenario *D* described in Example 2. We will adapt a more convenient notation for the generating simplices of the two triangles *A* and *B*. The first one will be denoted by $\sigma^{012}$ and the other one by $\sigma^{01^{\prime }2}$. The diamond *D* is obtained by gluing *A* and *B* along the $d_{1}$ face, i.e., the simplex $\sigma^{02}$. Again for ease of notation, the probabilities $p_{\sigma_{A}^{012}}^{ab}$ and $p_{\sigma_{B}^{01^{\prime }2}}^{a^{\prime }b^{\prime }}$ will be denoted by $p_{012}^{ab}$ and $p_{01^{\prime }2}^{a^{\prime }b^{\prime }}$, respectively. In this section, we will use the expectation coordinates introduced in Equations (3) and (4). These eight probabilities that are required to be non-negative are equivalent (up to an overall constant factor) to the inequalities

(16) $$\begin{array}{ccc} & & 1+{\left(-1\right)}^{a}{\bar{\sigma }}^{01}+{\left(-1\right)}^{b}{\bar{\sigma }}^{12}+{\left(-1\right)}^{a+b}{\bar{\sigma }}^{02}\geq 0,\; \end{array}$$

(17) $$\begin{array}{ccc} & & 1+{\left(-1\right)}^{a^{\prime }}{\bar{\sigma }}^{01^{\prime }}+{\left(-1\right)}^{b^{\prime }}{\bar{\sigma }}^{1^{\prime }2}+{\left(-1\right)}^{a^{\prime }+b^{\prime }}{\bar{\sigma }}^{02}\geq 0, \end{array}$$

for all $a,b,a^{\prime },b^{\prime }\in Z_{2}$.

**Proposition** **8.** *Let D be a diamond and ∂D denote its boundary. Then, a distribution p∈sDist(∂D) extends to $\tilde{p}\in \mathrm{sDist}\left(D\right)$ if and only if the CHSH inequalities are satisfied:*

(18) $${\left(-1\right)}^{a}{\bar{\sigma }}^{01}+{\left(-1\right)}^{b}{\bar{\sigma }}^{12}+{\left(-1\right)}^{a^{\prime }}{\bar{\sigma }}^{01^{\prime }}+{\left(-1\right)}^{b^{\prime }}{\bar{\sigma }}^{1^{\prime }2}\geq 0$$

*where $a,a^{\prime }b,b^{\prime }\in Z_{2}$ satisfying $a+b+a^{\prime }+b^{\prime }=1\;\mathrm{mod}\;2$.*

**Proof.**  Proof of this result is given in (Proposition 4.10 in [7]). We provide an exposition here for completeness. All of the coefficients that appear in Equations (16) and (17) are just ±1; to perform FM elimination, it suffices to sum up the inequalities where $\sigma^{02}$ has a positive and negative coefficient. For inequalities coming from the same triangle, this just yields that $-1\leq {\bar{\sigma }}^{ij}\leq 1$—we call such inequalities trivial. When we combine inequalities from different triangles, we obtain the inequalities in Equation (18). □

**Figure 5:**
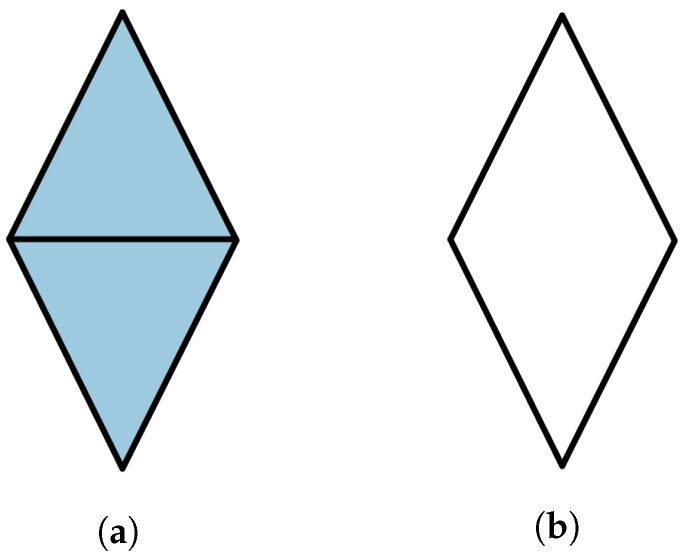
(**a**) The classical 4-disk is the diamond scenario. (**b**) FM elimination is interpreted geometrically as deleting an edge from a topological space.

**Remark** **2.** *We can interpret FM elimination geometrically as deleting an edge from a topological space; see Figure 5.*

In an abuse of terminology, we will sometimes say that we eliminate an edge *σ*, when what we actually mean is that we perform FM elimination on the corresponding expectation value $\bar{\sigma }$ that appears in the inequalities.

### 3.2. Extending to the Classical N-Disk

**Definition** **9.** *Let $\tau_{1},\cdots ,\tau_{N}$ denote the generating edges on the boundary of $D_{N}$. We define the N-circle inequalities by*

(19) $$\begin{array}{c} 0\leq N-2+\sum_{i=1}^{N}{\left(-1\right)}^{a_{i}}{\bar{\tau }}_{i} \end{array}$$

*where $\sum_{i=1}^{N}a_{i}=N+1\;\mathrm{mod}\;2$.*

**Example**  **4.** *Clearly, a triangle $\Delta^{2}$ is just a classical *3*-disk, and the *3*-circle inequalities come from Equation (4):*

$$p_{x}^{a}+p_{y}^{b}-p_{z}^{a+b+1}\geq 0.$$

*Note also that the diamond space is an example of a classical *4*-disk and the CHSH inequalities correspond to the *4*-circle inequalities.*

**Lemma**  **5.** *Consider a set of N-circle inequalities. We wish to apply FM elimination to a particular coordinate $\bar{z}$. The resulting inequalities are satisfied if the remaining coordinates ${\bar{\tau }}_{i}$ each satisfy $-1\leq {\bar{\tau }}_{i}\leq 1$.*

**Proof.**  Consider two inequalities where $\bar{z}$ appears with opposite signs

$$\begin{array}{ccc} & & 0\leq N-2+\bar{z}+\sum_{i=1}^{N-1}{\left(-1\right)}^{a_{i}}{\bar{\tau }}_{i}\\ & & 0\leq N-2-\bar{z}+\sum_{i=1}^{N-1}{\left(-1\right)}^{b_{i}}{\bar{\tau }}_{i} \end{array}$$

where $\sum_{i=1}^{N-1}a_{i}=N+1\;\mathrm{mod}\;2$ and $\sum_{i=1}^{N-1}b_{i}=N\;\mathrm{mod}\;2$. To perform FM elimination of $\bar{z}$, we add these inequalities together and observe that due to the conditions on $a_{i}$ and $b_{j}$, at least one other variable will cancel after summing. Let S⊂{1,⋯,N−1} index all variables that do *not* cancel. (Note that |S|≤N−2). The inequalities after summing become

(20) $$\begin{array}{c} 0\leq 2\left(N-2+\sum_{i\in S}{\left(-1\right)}^{a_{i}}{\bar{\tau }}_{i}\right), \end{array}$$

or equivalently

$$\begin{array}{c} 0\leq \left(N-2-|S|\right)+\sum_{i\in S}\left(1+{\left(-1\right)}^{a_{i}}{\bar{\tau }}_{i}\right). \end{array}$$

Using Equation (3), we see that these inequalities are satisfied if $-1\leq {\bar{\tau }}_{i}\leq 1$ for all i∈N: This condition gives us

$$\begin{array}{c} 0\leq \frac{N-2-|S|}{2}+\sum_{i\in S}p_{\tau_{i}}^{a_{i}}, \end{array}$$

where each term is non-negative since |S|≤N−2 and $0\leq p_{\tau_{i}}^{a_{i}}\leq 1$. Thus, the inequalities in Equation (20) are satisfied. □

**Lemma**  **6.** *Suppose we have a set of N-circle and M-circle inequalities that overlap on only a single variable $\bar{z}$. FM elimination of $\bar{z}$ yields a set of (N+M−2)-circle inequalities (plus trivial inequalities).*

**Figure 6:**
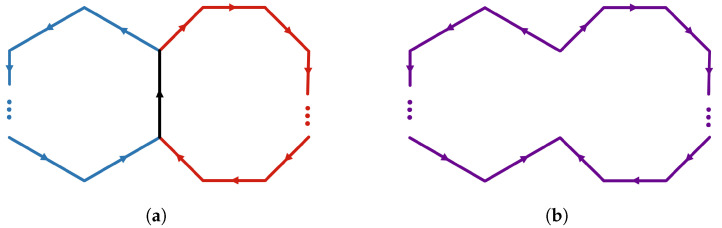
FM elimination of an edge (black) common to an *N*-circle and *M*-circle in (**a**) yields an inequality for the N+M−2-circle in (**b**).

**Proof.**  We begin by noting that if we sum up inequalities coming from the same set of circle inequalities, then by Lemma 5 we obtain trivial inequalities. Let us consider the other case where $\bar{z}$ comes from two different sets; see Figure 6. First, note that there are $2^{K-1}$ (K≥1) inequalities in a set of *K*-circle inequalities. Let $I_{M}^{\pm }$ index the *M*-circle inequalities where $\bar{z}$ has a positive (or negative) coefficient and observe that $|I_{M}^{\pm }|=2^{M-2}$. Similarly for $I_{N}^{\pm }$. FM elimination proceeds by summing up inequalities indexed by $\left(i,i^{\prime }\right)\in I_{M}^{+}\times I_{N}^{-}$ and $\left(j,j^{\prime }\right)\in I_{M}^{-}\times I_{N}^{+}$. This amounts to $2\times 2^{\left(M-2)+(N-2\right)}=2^{(N+M-2)-1}$ new inequalities, which is precisely the amount needed for a set of (N+M−2)-circle inequalities.

To find the precise form of these inequalities, let us consider explicitly two inequalities indexed by $\left(i,i^{\prime }\right)\in I_{M}^{-}\times I_{N}^{+}$. We denote the variables appearing in the *M*-circle and *N*-circle inequalities as $\tau_{j}$ (j=1,⋯,M) and $\tau_{k}^{\prime }$ (k=1,⋯,N), respectively, and denote $\bar{z}=\tau_{M}=\tau_{N}^{\prime }$. Summing the two inequalities, we obtain

$$\begin{array}{ccc} 0 & \leq & N-2+\bar{z}+\sum_{k=1}^{N-1}{\left(-1\right)}^{a_{k}}\tau_{k}^{\prime }+\left(M-2-\bar{z}+\sum_{j=1}^{M-1}{\left(-1\right)}^{b_{j}}\tau_{j}\right), \end{array}$$

where $\sum_{k=1}^{N-1}a_{k}=N+1\;\mathrm{mod}\;2$ and $\sum_{j=1}^{M-1}b_{j}=M\;\mathrm{mod}\;2$. This is equivalent to

(21) $$\begin{array}{ccc} 0 & \leq & \left(N+M-2\right)-2+\sum_{k=1}^{N-1}{\left(-1\right)}^{a_{k}}\tau_{k}^{\prime }+\sum_{j=1}^{M-1}{\left(-1\right)}^{b_{j}}\tau_{j} \end{array}$$

where $\sum_{k=1}^{n-1}a_{k}+\sum_{j=1}^{M-1}b_{j}=N+M+1\;\mathrm{mod}\;2$. Noting that N+M+1mod2=(N+M−2)+1mod2, this is precisely an (N+M−2)-circle inequality. A similar argument holds for $I_{M}^{+}\times I_{N}^{-}$, and this proves the result. □

We have the following corollary of Lemma 6:

**Corollary**  **4.** *Suppose we have a set of N-circle and *3*-circle inequalities that overlap on only a single variable $\bar{z}$. FM elimination of $\bar{z}$ yields a set of (N+1)-circle inequalities (plus trivial inequalities). See Figure 6.*

Next we apply these preliminary results to the classical *N*-disk scenario.

**Proposition**  **9.** *A distribution $p\in \mathrm{sDist}(\partial D_{N})$ extends to a distribution $\tilde{p}$ on $D_{N}$ if and only if the N-circle inequalities (and the trivial inequalities $-1\leq {\bar{\tau }}_{i}\leq 1$) are satisfied.*

**Proof.**  We consider a classical *N*-disk (e.g., see Figure 4), such that the edges on the boundary are labeled by $\tau_{i}$ (i=1,⋯,N) and those on the interior are denoted $z_{j}$ (j=1,⋯,N−3). For the first part of our proof, our strategy is to perform FM elimination successively on the interior edges $z_{j}$ beginning (it is well known that the order in which FM elimination is performed does not affect the final result; however, a “bad” ordering can lead to an explosion in intermediate inequalities to keep track of; see, e.g., [20]) with $z_{1}$ and ending in $z_{N-3}$. Consider the two classical 3-disks bounded by $\left\{\tau_{1},\tau_{N},z_{1}\right\}$ and $\left\{z_{1},\tau_{2},z_{2}\right\}$, respectively. By Corollary 4, FM elimination of ${\bar{z}}_{1}$ yields a set of 4-circle inequalities (plus trivial inequalities) together with the remaining inequalities in which ${\bar{z}}_{1}$ does not appear. For each successive application of FM for the edges $z_{j}$, we can apply Corollary 4. After N−3 iterations, we are left with an *N*-circle inequality, as well as trivial inequalities. This proves one direction. On the other hand, FM elimination guarantees that we can find a set of $\left\{z_{i}:\;i=1,\cdots ,N-3\right\}$ such that we can reverse this process and extend from the boundary to the *N*-order disk. □

### 3.3. Bouquet of Classical N-Disks

It is possible to extend Proposition 9 slightly by considering the union of *N* disks of varying size.

**Definition** **10.** *Let $C_{1}$ denote the simplicial set with a single generating *1*-simplex τ with the relation*

$$d_{0}\tau =d_{1}\tau .$$

*For N≥2, let $C_{N}$ denote the *1*-dimensional simplicial set consisting of the generating *1*-simplices $\tau_{1},\cdots ,\tau_{N}$ together with the identifying relations*

$$d_{i_{1}^{\prime }}\tau_{1}=d_{i_{2}}\tau_{2},\;d_{i_{2}^{\prime }}\tau_{2}=d_{i_{3}}\tau_{3}\;\cdots \;d_{i_{N}^{\prime }}\tau_{N}=d_{i_{1}}\tau_{1}$$

**Figure 9:**
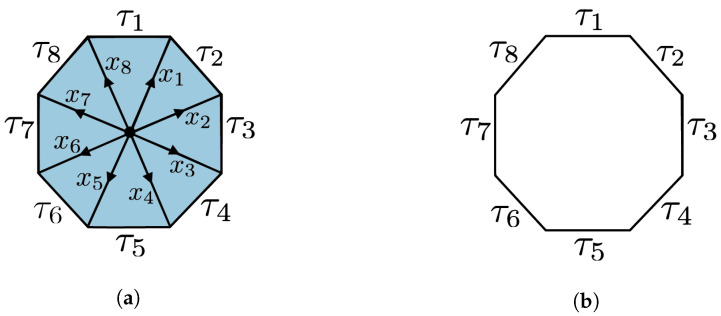
The *N*-cycle scenario (N=8) depicted in (**a**) is the cone of the *N*-circle scenario depicted in (**b**) consisting of the edges $\tau_{1},\cdots ,\tau_{8}$ on the boundary, and $x_{1},\cdots ,x_{8}$ in the interior.

*where $i_{k}\neq i_{k}^{\prime }\in 0,1$ for 1≤k≤N. We call $C_{N}$ the N-circle space, or simply the circle space when N=1; see Figure 9b. A circle of length N on a simplicial set X is given by an injective simplicial set map $C_{N}\to X$. We will also write $C_{N}$ for the image of this map.*

**Corollary**  **5.** *Let X be a two-dimensional simplicial set obtained by gluing $D_{N_{1}},\cdots ,D_{N_{k}}$ along a common edge, and let ∂X be the one-dimensional given by the boundary of X. Then, p∈sDist(∂X) extends to a distribution $\tilde{p}$ on X if and only if for every circle $C_{M_{j}}\subset \partial X$, where j=1,⋯,k2, we have that ${p|}_{C_{M_{j}}}$ satisfies the corresponding $M_{j}$-circle inequality.*

**Figure 7:**
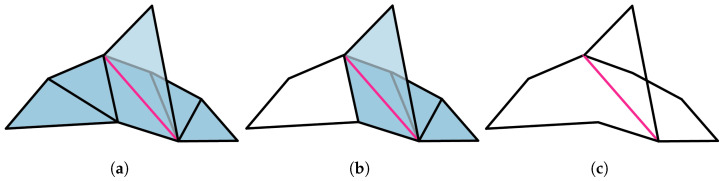
(**a**) A bouquet of classical *N*-disks is constructed by gluing each $D_{N_{i}}$ along a common edge (purple) that is a boundary edge of its initial triangle. (**b**) For each disk, we perform FM elimination, beginning with the interior edge of the terminal triangle. (**c**) We continue until only the common edge (purple) remains.

**Proof.**  For each $D_{N_{i}}$, its initial and terminal triangles, which we denote by $\sigma_{1}^{\left(i\right)}$ and $\sigma_{N_{i}-2}^{\left(i\right)}$, respectively, are distinguished via Equation (14). A bouquet *X* of classical *N*-disks is then constructed by gluing each $D_{N_{i}}$ along the single common edge *τ*, which we take to be either boundary edge of the initial triangle $\sigma_{1}^{\left(i\right)}$; see Figure 7a.

For each disk $D_{N_{i}}$ in *X*, we perform FM elimination on all interior edges, beginning with the interior edge of the terminal triangle and concluding with the interior edge of the initial triangle; see Figure 7b. The ordering of which disks FM elimination is applied to is arbitrary and does not affect the calculations. For each disk, we stop before eliminating the edge *τ*; see Figure 7c. By Proposition 9, this will result in *N*-circle inequalities (plus trivial inequalities), each corresponding to a circle of length $N_{i}$. Since *τ* appears in all *k* sets of circle inequalities, and it is the only edge in the intersection of these circles, then Lemma 6 applies. We will have k2 circles $C_{M_{j}}$, where j=1,⋯,k2. □

The extension result of Corollary 5 will be useful in proving Fine’s theorem in Section 4 for various types of scenarios.

**Example** **5.** *Bipartite $\left(m_{A},m_{B},d_{A},d_{B}\right)$ Bell scenarios consist of parties Alice and Bob performing one of $m_{A}$, $m_{B}$ measurements with one of $d_{A}$, $d_{B}$ outcomes, respectively. In [12] it was shown that by generalizing an argument due to Fine [2,3], the CHSH inequalities are also necessary and sufficient for this more general scenario.*

*A topological realization for (2,m,2,2) Bell scenario is given in Figure 8a. Note that this scenario is a special case of the flower scenario depicted in Figure 1. In Theorem 2, we will generalize Fine’s characterization of non-ontextual distributions to flower scenarios. The basic idea of our approach can be sketched in the case of (2,3,2,2) Bell scenario; see Figure 8b. In this case, we use the bouquet of *3*-disks depicted in Figure 8c. By Corollary 5, a distribution on the boundary Figure 8d extends to the whole space if and only if the 6=42 sets of *4*-circle inequalities are satisfied.*

## 4. Distributions on the *N*-Cycle Scenario and Beyond

The measurement space of the *N*-cycle scenario is a disk triangulated into *N* triangles as in Figure 9a.

**Definition** **11.** *Let ${\tilde{C}}_{N}$ denote the following simplicial set:*

- *Generating *2*-simplices: $\sigma_{1},\cdots ,\sigma_{N}$.*
- *Identifying relations:*
$$d_{i_{1}^{\prime }}\sigma_{1}=d_{i_{2}}\sigma_{2},\;d_{i_{2}^{\prime }}\sigma_{2}=d_{i_{3}}\sigma_{3}\;\cdots \;d_{i_{N}^{\prime }}\sigma_{N}=d_{i_{1}}\sigma_{1}$$
  *where $i_{k}\neq i_{k}^{\prime }\in 1,2$ for 1≤k≤N.*

*${\tilde{C}}_{1}$ has a single generating simplex σ with the identifying relation $d_{1}\sigma =d_{2}\sigma$.*

Topologically, ${\tilde{C}}_{N}$ is obtained from its boundary, which is circle consisting of *N* edges, by introducing a new point, the vertex in the middle, and coning off the boundary. This construction will be very useful in our analysis of simplicial distributions on two-dimensional measurement spaces.

Observe that ${\tilde{C}}_{N}$ is precisely the cone of $C_{N}$. In later sections, we will study the vertices of the polytope of simplicial distributions on this scenario and describe the Bell inequalities bounding the noncontextual distributions. Note that ${\tilde{C}}_{1}$ is a new scenario in the sense that it cannot be realized in the conventional picture of nonsignaling distributions, such as in the language of sheaf theory [5]. This is the smallest space on which a contextual simplicial distribution is defined.

### Topological Proof of Fine’s Theorem

The proof of Fine’s theorem for the CHSH scenario given in (Theorem 4.13 in [7]) relies on topological methods. Here, we show that these methods can be generalized to other interesting scenarios, including the *N*-cycle scenario and the flower scenario obtained by gluing cycle scenarios as in Figure 1.

**Lemma** **7.** *Let X be a simplicial set. The map*

$$\mathrm{dDist}\left(C\left(X\right)\right)\to Z_{2}^{|X_{0}|}$$

*that sends $\delta^{s}$ to ${\left(s\left(c,v\right)\right)}_{v\in X_{0}}$ is a bijection.*

**Proof.**  A deterministic distribution on $\Delta^{2}$ is given by an assignment (x,y,z)↦(a,b,c), such that a+b+c=0mod2. Therefore, in C(X), once the edges (c,v) are assigned an outcome, the remaining edges will be determined. □

**Lemma** **8.** *Let X be a simplicial set. Given a noncontextual distribution p∈sDist(C(X)), the restriction ${p|}_{C_{N}}$ to an N-circle $C_{N}\subset X$ satisfies the N-circle inequalities.*

**Proof.**  Let $D_{N}$ be a classical *N*-disc with $C_{N}$ as the boundary. Recall that we can think of ${\tilde{C}}_{N}$ as the cone of $C_{N}$. Note that ${\left(C_{N}\right)}_{0}={\left(D_{N}\right)}_{0}$; thus, using Lemma 7, we obtain that the map $\mathrm{dDist}(C\left(D_{N}\right)))\to \mathrm{dDist}({\tilde{C}}_{N})$ induced by the inclusion $C_{N}\to D_{N}$ is an isomorphism. We have the commutative diagram

(22) $$\begin{array}{ccc} D_{R}(\mathrm{dDist}\left(C\left(D_{N}\right)\right) & \overset{\Theta }{\;\to \;} & \mathrm{sDist}\left(C\left(D_{N}\right)\right)\\ ≅↓ & & ↓\\ D_{R}(\mathrm{dDist}(\left({\tilde{C}}_{N})\right) & \overset{\Theta }{\;\to \;} & \mathrm{sDist}({\tilde{C}}_{N}) \end{array}$$

The simplicial distribution *p* is noncontextual; thus, by Corollary 1, ${p|}_{{\tilde{C}}_{N}}$ is also noncontextual. Therefore, by Diagram (22), the distribution ${p|}_{{\tilde{C}}_{N}}$ can be extended to a distribution on $C(D_{N})$. In particular, ${p|}_{C_{N}}$ extended to a distribution on $D_{N}$. By Proposition 9 we obtain the result. □

**Proposition**  **10.** *A distribution $p\in \mathrm{sDist}({\tilde{C}}_{N})$ is noncontextual if and only if ${p|}_{C_{N}}$ satisfies the N-circle inequalities.*

**Figure 10:**
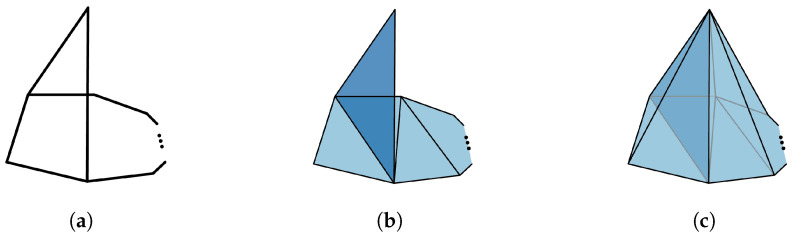
(**a**) *Z* is obtained from the *N*-circle by attaching two edges. (**b**) *W* is obtained from a classical *N*-disk by attaching a triangle. (**c**) *V* is obtained from *W* by attaching an *N*-cycle space.

**Proof.**  Forward direction is proved in Lemma 8. For the converse, we will need the following simplicial sets:

- $Z\subset {\tilde{C}}_{N}$ denotes the one-dimensional simplicial subset obtained by gluing two edges to $C_{N}$, as depicted in Figure 10a.
- *W* is obtained by gluing a triangle $\Delta^{2}$ to $D_{N}$, as in Figure 10b.
- *V* is the simplicial set obtained by gluing ${\tilde{C}}_{N}$ and *W* along *Z*, as in Figure 10c:
  (23) $$V={\tilde{C}}_{N}\cup_{Z}W.$$

**Figure 11:**
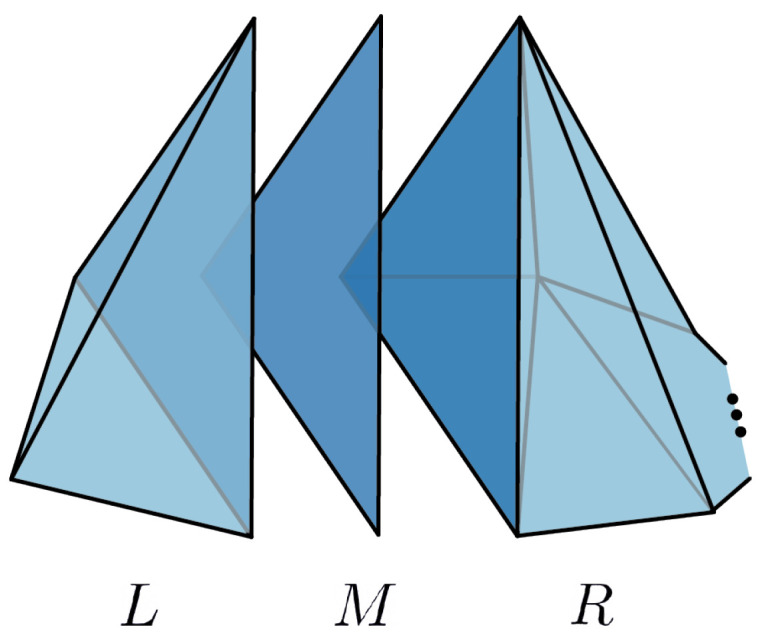
The space *V* partitioned into three simplicial subsets: *L*, *M*, and *R*.

Let *p* be a simplicial distribution on ${\tilde{C}}_{N}$, such that ${p|}_{C_{N}}$ satisfies the *N*-circle inequalities. By Proposition 9, we conclude that the restriction of *p* on the other two circles on *Z* also satisfies the circle inequalities. Therefore, Corollary 5 implies that ${p|}_{Z}$ extends to a simplicial distribution *q* on *W*. Since *p* and *q* match on *Z*, there is a simplicial distribution *P* on *V*, such that ${P|}_{{\tilde{C}}_{N}}=p$ and ${P|}_{W}=q$. Now, consider the following decomposition given in Figure 11:

$$V=L\cup_{M}R$$

where $L=\partial \Delta^{3}$ is the boundary of the tetrahedron, $M=\Delta^{2}$ is triangle. Let $\tilde{R}$ denote the simplicial subset of *R* obtained by omitting the bottom triangles. Note that $\tilde{R}$ is an (N−1)-cycle scenario. Let *Q* be the restriction of *P* on $L\cup_{M}\tilde{R}$. The simplicial distribution *Q* is noncontextual if and only if ${Q|}_{L}$ and ${Q|}_{\tilde{R}}$ are both noncontextual by (Gluing) Lemma 1. By (Proposition 4.12 in [7]), every simplicial distribution on $\partial \Delta^{3}$ is noncontextual. Therefore, it suffices to have ${Q|}_{\tilde{R}}$ be noncontextual to guarantee that *Q* is noncontextual. In fact, ${Q|}_{\tilde{R}}$ is the restriction of ${P|}_{R}$ on $\tilde{R}$; thus, by Proposition 9, we conclude that Q satisfies the circle inequalities on the lower circle of $\tilde{R}$. By induction on *N*, we conclude that ${Q|}_{\tilde{R}}$ is noncontextual. Finally, by Corollary 1, this implies that ${p=Q|}_{{\tilde{C}}_{N}}$ is noncontextual. □

Combining this result with Proposition 9 gives the following result, which will be used in the generalization of Proposition 10 to the flower scenario.

**Corollary**  **6.** *A distribution p on ${\tilde{C}}_{N}$ is noncontextual if and only if it extends to a distribution on ${\tilde{C}}_{N}\cup_{C_{N}}D_{N}$.*

Taking N=3 in Corollary 6 gives us a sufficient and necessary condition when a distribution on the boundary of a triangle can be extended to a distribution on the triangle. Thus, we obtain a useful result that characterizes the image of the map $f:\mathrm{sDist}\left(X\right)\to \mathrm{sDist}(X^{\left(1\right)})$ introduced in (7). (We remark that the following result still holds when the restriction that $\partial \sigma_{i}$ does not contain nondegenerate edges is removed in Proposition 4).

**Corollary**  **7.** *Let X be a two-dimensional simplicial set. A distribution $q\in \mathrm{sDist}(X^{\left(1\right)})$ is in the image of the map f in (7) if and only if $\left(q_{d_{0}\sigma }^{0},q_{d_{1}\sigma }^{0},q_{d_{2}\sigma }^{0}\right)$ satisfies the *3*-circle inequality for all $\sigma \in X_{2}$.*

To generalize Proposition 10 to the flower scenario, we need a stronger version of the Gluing lemma.

**Lemma** **9.** *Let $X=\cup_{i=1}^{m}A_{i}$, such that $A_{i}\cap A_{j}=\Delta^{n}$ for every i≠j. Then, p∈sDist(X) is noncontextual if and only if ${p|}_{A_{i}}$ is noncontextual for every 1≤i≤m.*

**Proof.**  Follows by induction and Lemma 1. □

The flower scenario is obtained by gluing lines at their end points. Let $L_{N}$ denote the simplicial set consisting of the generating 1-simplices $\tau_{1},\cdots ,\tau_{N}$ together with the identifying relations

$$d_{i_{1}^{\prime }}\tau_{1}=d_{i_{2}}\tau_{2},\;d_{i_{2}^{\prime }}\tau_{2}=d_{i_{3}}\tau_{3}\;\cdots \;d_{i_{N-1}^{\prime }}\tau_{N-1}=d_{i_{N}}\tau_{N}$$

where $i_{k}\neq i_{k}^{\prime }\in \{0,1\}$. Topologically, this simplicial set represents a line of length *N.*

**Figure 12:**
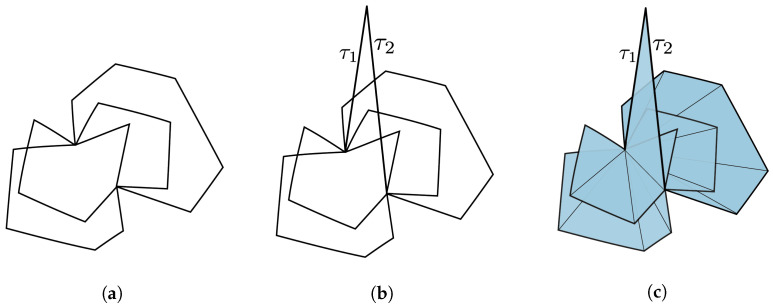
(**a**) *X* is obtained by gluing lines at their end points. (**b**) *Z* comes with an additional piece consisting of two edges. (**c**) *W* is obtained by filling the circles in *Z* with classical disks.

**Definition** **12.** *Let $X(N_{1},\cdots ,N_{k})$ denote the simplicial set obtained by gluing the lines $L_{N_{1}},\cdots ,$ $L_{N_{k}}$ at their boundary, i.e., the two terminal points v and w; see Figure 12a. We will call the cone of $X(N_{1},\cdots ,N_{k})$ a flower scenario.*

**Theorem** **2.** *Let C(X) denote the flower scenario where $X=X(N_{1},\cdots ,N_{k})$. A distribution p∈sDist(C(X)) is noncontextual if and only if for every circle $C_{N}$ on X the restriction ${p|}_{C_{N}}$ satisfies the N-circle inequalities.*

**Proof.**  Forward direction follows from Lemma 8. For the converse, we introduce the following simplicial sets:

- Z⊂C(X) denotes the one-dimensional simplicial set obtained by gluing two edges $\tau_{1}$ and $\tau_{2}$ to *X*, as depicted in Figure 12b.
- *W* is obtained by filling in the circles in *Z* by classical disks, as in Figure 12c.
- Gluing C(X) with *W* along the intersection *Z*, we obtain
  $$V=C\left(X\right)\cup_{Z}W.$$

**Figure 13:**
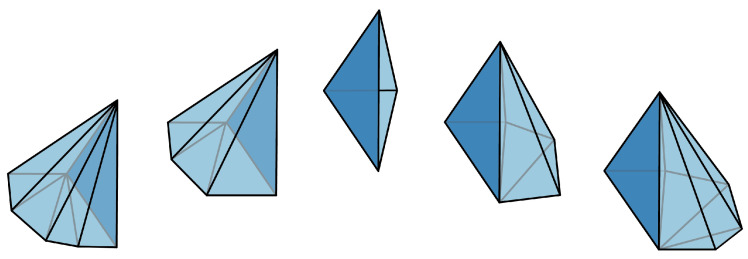
$V_{i}$ for i=1,⋯,5.

Let *p* be a simplicial distribution on C(X) satisfying the circle inequalities for every circle in *X*. Moreover, by Proposition 9, the distribution *p* also satisfies the circle inequalities for the remaining circles in the larger space *Z*, since on these circles, the distribution extends to classical disks contained in C(X). Then, Corollary 5 implies that ${p|}_{Z}$ extends to a simplicial distribution *q* on *W*. The two distributions *p* and *q* give a distribution *P* on *V*. Now, we define the following simplicial subsets of *V*:

- *M* denotes the triangle in Figure 12c with two of the edges given by $\tau_{1}$ and $\tau_{2}$.
- ${\tilde{V}}_{i}$ is obtained by gluing $C(L_{N_{i}})$ and *M* along $\tau_{1}$ and $\tau_{2}$.
- $V_{i}$ is obtained by gluing ${\tilde{V}}_{i}$ and the classical disk contained in *W,* whose boundary coincides with $\partial {\tilde{V}}_{i}$; see Figure 13.

Note that $M={\tilde{V}}_{i}\cap {\tilde{V}}_{j}=V_{i}\cap V_{j}$ for distinct i,j. In addition, we obtain another decomposition of *V*, as given in Figure 13:

$$V=V_{1}\cup_{M}V_{1}\cup_{M}\cdots \cup_{M}V_{k}.$$

By Corollary 6, the simplicial distribution ${P|}_{{\tilde{V}}_{i}}$ is noncontextual, since it is the restriction of ${P|}_{V_{i}}$. Therefore, by Lemma 9, the distribution ${P|}_{{\tilde{V}}_{1}\cup_{M}{\tilde{V}}_{2}\cup_{M}\cdots \cup_{M}{\tilde{V}}_{k}.}$ is noncontextual, so by Corollary 1, the restriction ${p=P|}_{\left(X\right)}$ is noncontextual. □

## 5. Collapsing Measurement Spaces

In this section, we study the effect of collapsing simplices in the measurement space. This method is very effective in describing the vertices of the polytope of simplicial distributions.

Let us begin with the simplest case of collapsing a single edge to a point. Recall that $\Delta^{1}$ is the simplicial set representing an edge. It has a single generating simplex in dimension 1 denoted by $\sigma^{01}$. A point is represented by the simplicial set $\Delta^{0}$. Its *n*-simplices are given by $c_{n}$ obtained by applying the $s_{0}$ degeneracy map *n*-times: $s_{0}\cdots s_{0}\left(\sigma^{0}\right)=\sigma^{0\cdots 0}$. Collapsing an edge to a point can be represented by a simplicial set map

$$\pi :\Delta^{1}\to \Delta^{0}$$

that sends the generating simplex $\sigma^{01}$ to the degenerate simplex $\sigma^{00}$. Now, applying the cone construction to this map, we obtain a simplicial set map

$$C\pi :C(\Delta^{1})\to C(\Delta^{0})$$

Recall from Figure 2a that $C(\Delta^{1})$ can be identified with a triangle whose generating simplex is given by $\left(c,\sigma^{01}\right)$. A similar topological intuition works for $C(\Delta^{0})$. It represents an edge whose generating simplex is $\left(c,\sigma^{0}\right)$. From this, we can work out the map Cπ as follows: The generating simplex $\left(c,\sigma^{01}\right)$ is mapped to $\left(c,s_{0}\sigma^{0}\right)$, since *π* sends $\sigma^{01}$ to the degenerate simplex $s_{0}\sigma^{0}$. By the simplicial structure of the cone described in Definition 4, we have

(24) $$\left(c,s_{0}\sigma^{0}\right)=s_{1}\left(c,\sigma_{0}\right).$$

As we have seen in Section 2.2, the map Cπ between the cone spaces induces a map between the associated simplicial distributions

$${\left(C\pi \right)}^{*}:\mathrm{sDist}\left(C\left(\Delta^{0}\right)\right)\to \mathrm{sDist}\left(C\left(\Delta^{1}\right)\right)$$

**Figure 14:**
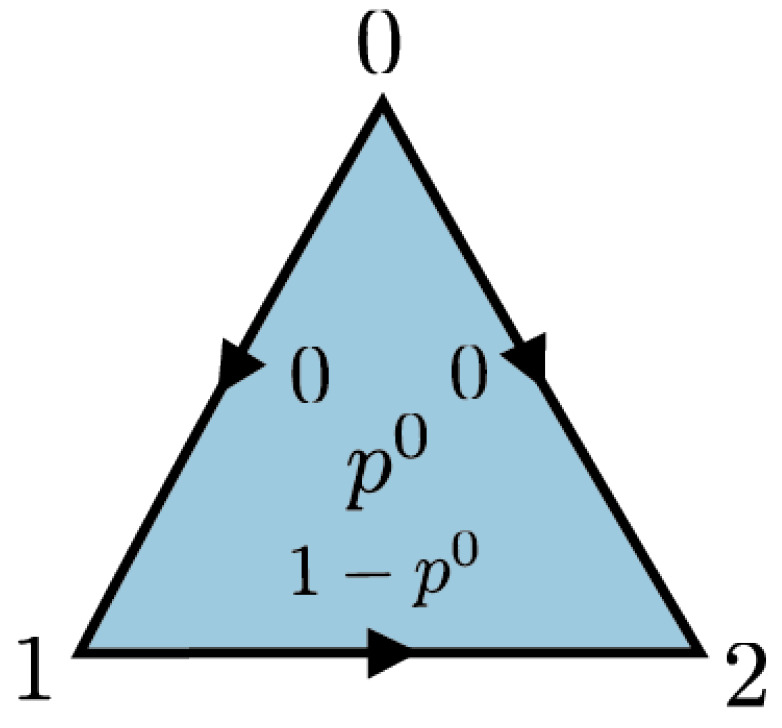
Collapsed distribution on the triangle.

A simplicial distribution $p\in \mathrm{sDist}(C\left(\Delta^{0}\right))$ is determined by $p_{\left(c,\sigma^{0}\right)}\in D_{R}\left(Z_{2}\right)$. Let *q* denote the image of *p*, i.e., $q={\left(C\pi \right)}^{*}\left(p\right)$. Then, *q* will be determined by $q_{\left(c,\sigma^{01}\right)}$, a distribution on $Z_{2}^{2}$. It is given as follows; see Figure 14:

(25) $$\begin{array}{cc} q_{\left(c,\sigma^{01}\right)}^{ab} & =p_{\pi (c,\sigma^{01})}^{ab}\\ \; & =p_{\left(c,s_{0}\sigma^{0}\right)}^{ab}\\ \; & =p_{s_{1}\left(c,\sigma^{0}\right)}^{ab}\\ \; & ={\left(D_{R}\left(s_{1}\right)\left(p_{\left(c,\sigma^{0}\right)}\right)\right)}^{ab}\\ \; & =\left\{\begin{array}{cc} p_{\left(c,\sigma^{0}\right)}^{0} & \left(a,b)=(0,0\right)\\ 1-p_{\left(c,\sigma^{0}\right)}^{0} & \left(a,b)=(1,0\right)\\ 0 & \mathrm{otherwise}, \end{array}\right. \end{array}$$

where in the first line, we use q=p∘Cπ; in the second line, the definition of Cπ; in the third line, Equation (24); in the fourth line, compatibility of *p* with the simplicial structure; and in the fifth line, the definition of $D_{R}\left(s_{1}\right)$. We will refer to this distribution as a *collapsed distribution* on the triangle.

Next, we consider the general case. Let *X* be a one-dimensional simplicial set and *σ* denote a nondegenerate 1-simplex, such that

(26) $$d_{0}\left(\sigma \right)\neq d_{1}\left(\sigma \right).$$

**Figure 15:**
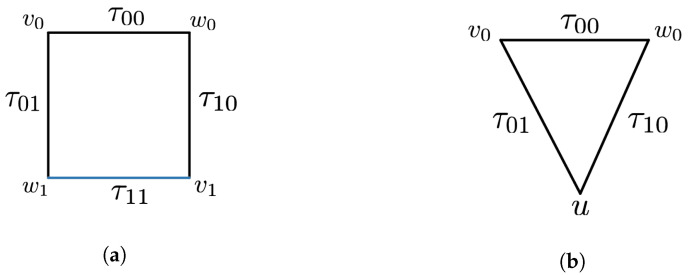
The 3-circle (**b**) is obtained by collapsing the edge $\tau_{11}$ (blue) in (**a**).

We will write X/σ for the simplicial set obtained by collapsing (the simplicial set X/σ is the quotient of *X* by the simplicial subset generated by *σ* in the sense of (Section 5.3 in [7])) this edge. More formally, X/σ consists of the same generating simplices as *X*, except *σ* and the simplicial relations are inherited from *X*. We will write π:X→X/σ for the collapsing map as before. We also have Cπ:C(X)→C(X/σ), which collapses the triangle obtained as the cone of *σ*. In Figure 15, we represent the collapsing maps $\pi :C_{4}\to C_{3}$ between two circle scenarios.

**Lemma** **10.** *For a collapsing map π:X→X/σ, the following properties hold.*

*1.*  *The map*
$${\left(C\pi \right)}^{*}:\mathrm{sDist}\left(C\left(X/\sigma \right)\right)\to \mathrm{sDist}\left(C\left(X\right)\right)$$
  is injective. Moreover, a distribution q∈sDist(C(X)) lies in the image of ${\left(C\pi \right)}^{*}$ if and only if $q_{\left(c,\sigma \right)}$ is a collapsed distribution.
*2.*  *The map*
$${\left(C\pi \right)}^{*}:\mathrm{dDist}\left(C\left(X/\sigma \right)\right)\to \mathrm{dDist}\left(C\left(X\right)\right)$$
  is injective. Moreover, a deterministic distribution $\delta^{t}\in \mathrm{dDist}\left(C\left(X\right)\right)$ lies in the image of ${\left(C\pi \right)}^{*}$ if and only if $t_{\left(c,\sigma \right)}\in \left\{\left(0,0\right),\left(1,0\right)\right\}$.

**Proof.**  The surjectivity of ${\left(C\pi \right)}_{n}$ for every n≥0 implies the injectivity of ${\left(C\pi \right)}^{*}$ in both cases. For p∈sDist(C(X)), the definition of the collapsing map implies that for every generating 1-simplex τ≠σ in *X*, we have ${\left(C\pi \right)}^{*}{\left(p\right)}_{\left(c,\tau \right)}=p_{\left(c,\tau \right)}$. Using Equation (25), we obtain that ${\left(C\pi \right)}^{*}{\left(p\right)}_{\left(c,\sigma \right)}$ is a collapsed distribution. By part (1), a deterministic distribution $\delta^{t}\in \mathrm{dDist}\left(C\left(X\right)\right)$ lies in the image of ${\left(C\pi \right)}^{*}$ if and only if $\delta^{t_{\left(c,\sigma \right)}}$ is a collapsed distribution. This is equivalent to $t_{\left(c,\sigma \right)}\in \left\{\left(0,0\right),\left(1,0\right)\right\}$. □

**Theorem** **3.** Let X be a one-dimensional simplicial set, and π:X→X/σ denote a collapsing map. For p∈sDist(C(X/σ)) and $q={\left(C\pi \right)}^{*}\left(p\right)$, the following holds.

*1.*  *p is contextual if and only if q is contextual.*
*2.*  *p is strongly contextual if and only if q is strongly contextual.*
*3.*  *p is a vertex if and only if q is a vertex.*
*4.*  *p is deterministic distribution if and only if q is deterministic distribution.*

**Proof.**  Part (1): Proposition 2 implies that if *q* is contextual, then *p* is contextual. For the converse, assume that *q* is noncontextual. Then, there exists $d=\sum_{i=1}^{n}d\left(s^{i}\right)\delta^{s^{i}}\in D_{R}\left(\mathrm{dDist}(C(X))\right)$, where $d(s^{i})\neq 0$, such that for every simplex *τ* in C(X), we have

(27) $$q_{\tau }=\sum_{i=1}^{n}d\left(s^{i}\right)\delta^{s_{\tau }^{i}}.$$

By part (1) of Lemma 10, the distribution $q_{\left(c,\sigma \right)}$ is collapsed. By Equation (27), we conclude that $s_{\left(c,\sigma \right)}^{i}\in \left\{\left(0,0\right),\left(1,0\right)\right\}$ for every i=1,⋯,n. Therefore, by part (2) of Lemma 10, we have $\delta^{r^{i}}\in \mathrm{dDist}\left(C\left(X/\sigma \right)\right)$, such that ${\left(C\pi \right)}^{*}\left(\delta^{r^{i}}\right)=\delta^{s^{i}}$. We define $\tilde{d}\in D_{R}\left(\mathrm{sDist}(C(X/\sigma ))\right)$ by $\sum_{i=1}^{n}d\left(r^{i}\right)\delta^{r^{i}}$. Then, $D_{R}\left({\left(C\pi \right)}^{*}\right)\left(\tilde{d}\right)=d$. Therefore, using the commutativity of Diagram (5) for f=Cπ, we obtain that

$${\left(C\pi \right)}^{*}\left(\Theta \left(\tilde{d}\right)\right)=\Theta \left(D_{R}\left({\left(C\pi \right)}^{*}\right)\left(\tilde{d}\right)\right)=\Theta \left(d\right)={\left(C\pi \right)}^{*}\left(p\right)$$

Since ${\left(C\pi \right)}^{*}$ is injective $\Theta (\tilde{d})=p$, which means that *p* is noncontextual.

Part (2): If q is strongly contextual then p is strongly contextual by (Lemma 5.19, part (1) in [14]). For the converse, assume that s∈supp(q). Then, $q_{\left(c,\sigma \right)}\left(s\left(c,\sigma \right)\right)\neq 0$. By part (1) of Lemma 10, the distribution $q_{\left(c,\sigma \right)}$ is collapsed. We conclude that $s_{\left(c,\sigma \right)}\in \left\{\left(0,0\right),\left(1,0\right)\right\}$. By part (2) of Lemma 10, there exists $\delta^{r}\in \mathrm{dDist}(X/\sigma )$, such that ${\left(C\pi \right)}^{*}\left(\delta^{r}\right)=\delta^{s}$. To show that r∈supp(p), it is enough to prove that for every nondegenerate simplex $\tau \in {\left(X/\sigma \right)}_{1}$, we have $p_{\left(c,\tau \right)}\left(r_{\left(c,\tau \right)}\right)\neq 0$. Note that since τ≠σ, we have

$$p_{\left(c,\tau \right)}=q_{\left(c,\tau \right)}\;\;\mathrm{and}\;\;\delta_{\left(c,\tau \right)}^{r}={\left(C\pi \right)}^{*}{\left(\delta^{r}\right)}_{\left(c,\tau \right)}=\delta_{\left(c,\tau \right)}^{s}.$$

Therefore, $p_{\left(c,\tau \right)}\left(r_{\left(c,\tau \right)}\right)\neq 0$ since s∈supp(q).

Part (3): According to (Corollary 5.16 in [14]), every vertex in the preimage of *q* under ${\left(C\pi \right)}^{*}$ is a vertex in sDist(CX/σ). Because of the injectivity of ${\left(C\pi \right)}^{*}$, this preimage contains just *P*; thus, *p* is a vertex. For the converse, suppose we have distributions $q_{1},q_{2}\in \mathrm{sDist}\left(CX\right)$ and 0<α<1, such that

$$q=\alpha q^{1}+\left(1-\alpha \right)q^{2}$$

By part (1) of Lemma 10, the distribution $q_{\left(c,\sigma \right)}$ is a collapsed distribution; thus, $q_{\left(c,\sigma \right)}^{1}$ and $q_{\left(c,\sigma \right)}^{2}$ are also collapsed. Again, by part (1) of Lemma 10, there exists ${\tilde{q}}^{1},{\tilde{q}}^{2}\in \mathrm{sDist}\left(C\left(X/\sigma \right)\right)$, such that ${\left(C\pi \right)}^{*}\left({\tilde{q}}^{i}\right)=q^{i}$, which implies that

$$q=\alpha {\left(C\pi \right)}^{*}\left({\tilde{q}}^{1}\right)+\left(1-\alpha \right){\left(C\pi \right)}^{*}\left({\tilde{q}}^{2}\right)={\left(C\pi \right)}^{*}\left(\alpha {\tilde{q}}^{1}+\left(1-\alpha \right){\tilde{q}}^{2}\right)$$

The map ${\left(C\pi \right)}^{*}$ is injective; therefore, $p=\alpha {\tilde{q}}^{1}+\left(1-\alpha \right){\tilde{q}}^{2}$. Since *p* is a vertex and 0<α<1, we conclude that ${\tilde{q}}^{1}={\tilde{q}}^{2}$. Therefore, $q^{1}=q^{2}$.

Part (4): By (Proposition 5.14 in [14]), every deterministic distribution is a vertex in the polytope of simplicial distributions. Thus, we can characterize deterministic distributions as the only noncontextual vertices. Then, we obtain this result from parts (1) and (3). □

**Corollary** **8.** A distribution p∈sDist(C(X/σ)) is a contextual vertex if and only if ${\left(C\pi \right)}^{*}\left(p\right)$ is a contextual vertex.

**Proof.**  This follows directly from parts (1) and (3) of Theorem 3. □

**Remark** **3.** *The conclusions of Theorem 3 and Corollary 8 hold for more general kinds of collapsing maps obtained by collapsing a set of edges in sequence.*

### 5.1. Application to Bell Inequalities

Consider the collapsing map π:X→X/σ and suppose that the Bell inequalities for the scenario C(X) are known. By part (1) of Theorem 3, a simplicial distribution p∈sDist(C(X/σ)) is noncontextual if and only if $q={\left(C\pi \right)}^{*}\left(p\right)\in \mathrm{sDist}\left(C\left(X\right)\right)$ is noncontextual. This is equivalent to the condition that *q* satisfies the Bell inequalities for the scenario C(X). From these Bell inequalities, we can extract those for the collapsed scenario C(X/σ).

Let us illustrate how the collapsing technique can be applied to cycle scenarios. Let $\pi :C_{4}\to C_{3}$ denote the map that collapses one of the edges in the 4-circle space, as in Figure 15.

By Proposition 4, a simplicial distribution $p\in \mathrm{sDist}({\tilde{C}}_{3})$ is specified by a tuple

$$p=(p_{\left(c,v_{0}\right)},p_{\tau_{00}},p_{\left(c,w_{0}\right)},p_{\tau_{10}},p_{\left(c,u\right)},p_{\tau_{01}})$$

where each entry is a distribution on $Z_{2}$. On the other hand, a simplicial distribution *q* on ${\tilde{C}}_{4}$ is specified by a tuple

$$\left(q_{\left(c,v_{0}\right)},q_{\tau_{00}},q_{\left(c,w_{0}\right)},q_{\tau_{10}},q_{\left(c,v_{1}\right)},q_{\tau_{11}},q_{\left(c,w_{1}\right)},q_{\tau_{01}}\right)$$

Then, the image of *p* under the map ${\left(C\pi \right)}^{*}$ gives us $q=(p_{\left(c,v_{0}\right)},p_{\tau_{00}},p_{\left(c,w_{0}\right)},p_{\tau_{10}},p_{\left(c,u\right)},1,p_{\left(c,u\right)},$$p_{\tau_{01}})$. This latter simplicial distribution is noncontextual if and only if it satisfies the 4-circle inequalities (see Equation (12)):

$$\begin{array}{c} 0\leq p_{\tau_{00}}+p_{\tau_{10}}+1-p_{\tau_{01}}\leq 2\\ 0\leq p_{\tau_{00}}+p_{\tau_{10}}-1+p_{\tau_{01}}\leq 2\\ 0\leq p_{\tau_{00}}-p_{\tau_{10}}+1+p_{\tau_{01}}\leq 2\\ 0\leq -p_{\tau_{00}}+p_{\tau_{10}}+1+p_{\tau_{01}}\leq 2. \end{array}$$

Half of these inequalities are trivial, so this set of inequalities is equivalent to

$$\begin{array}{c} p_{\tau_{00}}+p_{\tau_{10}}-p_{\tau_{01}}\leq 1\\ p_{\tau_{00}}+p_{\tau_{10}}+p_{\tau_{01}}\geq 1\\ p_{\tau_{00}}-p_{\tau_{10}}+p_{\tau_{01}}\leq 1\\ -p_{\tau_{00}}+p_{\tau_{10}}+p_{\tau_{01}}\leq 1 \end{array}$$

which constitute the nontrivial 3-circle inequalities.

**Figure 16:**
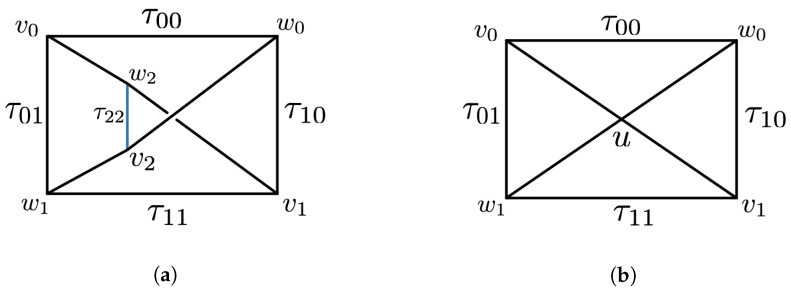
(**a**) The complete bipartite graph $K_{3,3}$. (**b**) The graph obtained by collapsing the edge $\tau_{22}$ to the vertex *u*.

Now, we will apply this technique to find the Bell inequalities for the cone of the one-dimensional space given in Figure 16b. This space will be our collapsed space X/σ. The one-dimensional simplicial set *X* is the complete bipartite graph $K_{3,3}$ given in Figure 16a. We denote the edge from $v_{i}$ to $w_{j}$ by $\tau_{ij}$. Note that the measurement space C(X) represents the (3,3,2,2) Bell scenario. It has three kinds of Bell inequalities: (1) trivial, (2) circle inequalities, and (3) Froissart inequalities [13]. We are interested in the latter type. An example of Froissart inequalities is the following (see Equation (21) in [21]):

$$\begin{array}{ccc} & & p_{\left(c,v_{0}\right)}^{0}+p_{\left(c,w_{0}\right)}^{0}-p_{\left(c,\tau_{00}\right)}^{00}-p_{\left(c,\tau_{01}\right)}^{00}-p_{\left(c,\tau_{02}\right)}^{00}\\ & & \;\;-p_{\left(c,\tau_{10}\right)}^{00}-p_{\left(c,\tau_{20}\right)}^{00}-p_{\left(c,\tau_{11}\right)}^{00}+p_{\left(c,\tau_{12}\right)}^{00}+p_{\left(c,\tau_{21}\right)}^{00}\geq -1 \end{array}$$

It will be convenient for us to convert this inequality to one that only contains distribution on edges. For this, we will use Equation (2). This substitution gives us the following inequality:

(28) $$\begin{array}{cc} p_{\tau_{12}}^{0}+p_{\tau_{21}}^{0}-p_{\tau_{00}}^{0}-p_{\tau_{01}}^{0} & -p_{\tau_{02}}^{0}-p_{\tau_{10}}^{0}-p_{\tau_{20}}^{0}-p_{\tau_{11}}^{0}\\ & -p_{\left(c,v_{0}\right)}^{0}-p_{\left(c,w_{0}\right)}^{0}-p_{\left(c,v_{1}\right)}^{0}-p_{\left(c,w_{1}\right)}^{0}\geq -6 \end{array}$$

We observe that the only edges that do not appear in this inequality are $\left(c,v_{2}\right),\left(c,w_{2}\right),\tau_{22}$. Applying the symmetries of X—more precisely, the automorphism group of the graph $K_{3,3}$—we can obtain nine distinct such inequalities in which the edges $\left(c,v_{i}\right),\left(c,w_{j}\right),\tau_{ij}$ do not appear, where i,j∈{0,1,2}. For example, for i=1,j=2, we have

(29) $$\begin{array}{cc} p_{\tau_{02}}^{0}+p_{\tau_{10}}^{0}-p_{\tau_{00}}^{0}-p_{\tau_{01}}^{0} & -p_{\tau_{22}}^{0}-p_{\tau_{21}}^{0}-p_{\tau_{20}}^{0}-p_{\tau_{11}}^{0}\\ & -p_{\left(c,v_{0}\right)}^{0}-p_{\left(c,w_{0}\right)}^{0}-p_{\left(c,v_{2}\right)}^{0}-p_{\left(c,w_{1}\right)}^{0}\geq -6 \end{array}$$

Note that these nine inequalities are in distinct orbits under the action of dDist(C(X)), since different edges appear in every one of them (see Example 3). We can find the number of Froissart inequalities in every orbit. A deterministic distribution $\delta^{s}$ fixes the inequality (28) if and only if ${\left(\delta_{\tau }^{s}\right)}^{0}=1$ for every edge *τ* that appears in the inequality. In this case, $\delta^{s}$ is the identity, i.e., ${\left(\delta_{\sigma }^{s}\right)}^{00}=1$ for every triangle *σ* in C(X). This implies that the size of the orbit of this inequality is equal to $\left|\mathrm{dDist}\left(C\left(X\right)\right)\right|=2^{6}=64$. The same counting argument works for the rest of the nine inequalities. Therefore, there are 9·64=576 Bell inequalities of this type.

Next, we apply our collapsing technique to generate a new Bell inequality, i.e., one that is not a circle inequality for the cone of the scenario given in Figure 16b). We will use the following collapsing map π:X→X/σ where $\sigma =\tau_{22}$. For a given circle on X, there is a corresponding circle inequality. Every such inequality will appear as a Bell inequality in the collapsed scenario X/σ if the corresponding circle does not contain the collapsed edge $\tau_{22}$. If the circle contains $\tau_{22}$, the resulting Bell inequality will be a circle inequality of a size one less as in Definition 9. Given a simplicial distribution p∈sDist(X/σ), the image $q={\left(C\pi \right)}^{*}\left(p\right)$ satisfies

$$q_{\tau_{22}}^{0}=1\;\;\mathrm{and}\;\;q_{\left(c,w_{2}\right)}^{0}=q_{\left(c,v_{2}\right)}^{0}=p_{\left(c,u\right)}^{0}.$$

Substituting this in (29), we obtain the following Bell inequality of the scenario X/σ:

(30) $$\begin{array}{c} p_{\tau_{02}}^{0}+p_{\tau_{10}}^{0}-p_{\tau_{00}}^{0}-p_{\tau_{01}}^{0}-p_{\tau_{21}}^{0}-p_{\tau_{20}}^{0}-p_{\tau_{11}}^{0}-p_{\left(c,v_{0}\right)}^{0}-p_{\left(c,w_{0}\right)}^{0}-p_{\left(c,u\right)}^{0}-p_{\left(c,w_{1}\right)}^{0}\geq -5 \end{array}$$

This inequality is a new Bell inequality, that is, it is not a circle inequality, and it belongs to a scenario that is not a Bell scenario. The latter observation implies that going beyond Bell scenarios can produce simpler Bell inequalities that are not circle inequalities; see [22].

**Remark**  **4.** *Let X be a one-dimensional simplicial set. Consider a Bell inequality for the cone scenario C(X) expressed in the edge coordinates (see Proposition 4). Then, in the known examples, the edges that appear with nontrivial coefficients in this Bell inequality form a loop (i.e., a circle with possible self-intersections) on X. It is a curious question whether this observation holds for every one-dimensional X. If so, it gives a topological restriction on the form of possible Bell inequalities, hence a nice structural result in contextuality.*

### 5.2. Detecting Contextual Vertices

In this section, the 1-circle $C_{1}$ will play a fundamental role in detecting contextual vertices in the scenarios of interest. Let *τ* denote the generating 1-simplex of $C_{1}$. A simplicial distribution $p\in \mathrm{sDist}({\tilde{C}}_{1})$ is specified by

$$(p_{\left(c,\tau \right)}^{00},p_{\left(c,\tau \right)}^{01},p_{\left(c,\tau \right)}^{10},p_{\left(c,\tau \right)}^{11}),$$

where

$$p_{\left(c,\tau \right)}^{ab}\in \left[0,1\right],\;\;\sum_{a,b}p_{\left(c,\tau \right)}^{ab}=1\;\;\;\mathrm{and}\;\;\;p_{\left(c,\tau \right)}^{00}+p_{\left(c,\tau \right)}^{01}=p_{\left(c,\tau \right)}^{00}+p_{\left(c,\tau \right)}^{11}.$$

**Figure 17:**
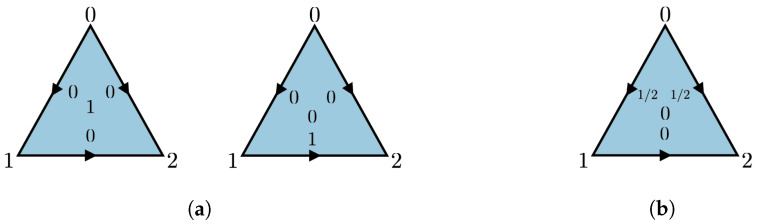
The 1-cycle scenario is obtained by identifying the edges (0,1) and (1,2) of a triangle. Deterministic (**a**) and contextual (**b**) vertices of the 1-cycle scenario.

This implies that $p_{\left(c,\tau \right)}^{01}=p_{\left(c,\tau \right)}^{11}$. Therefore, the polytope $\mathrm{sDist}\left({\tilde{C}}_{1}\right)\subset R^{3}$ is a triangle with two deterministic vertices and a unique contextual vertex $p_{-}$, given in Figure 17.

The following example shows how to obtain a contextual vertex in an arbitrary cycle scenario from the contextual vertex in Figure 17b using the collapsing technique.

**Example**  **6.** Let $\pi :C_{N}\to C_{1}$ denote the map that collapses $\tau_{2},\cdots ,\tau_{N}$ in the N-circle scenario. We will write $\tau =\tau_{1}$ for notational simplicity. Let $q={\left(C\pi \right)}^{*}\left(p_{-}\right)$ where p− is the unique contextual vertex of $\mathrm{sDist}({\tilde{C}}_{1})$. We have

$$q_{\left(c,d_{i}\left(\tau \right)\right)}^{0}={\left(p_{-}\right)}_{\left(c,d_{i}\left(\tau \right)\right)}^{0}=1/2$$

*(see Figure 17b). Then, by Equation (25), for $\tau^{\prime }=\tau_{2}$ and $\tau_{N}$, we have*

$$q_{\left(c,\tau^{\prime }\right)}=p_{+}.$$

*We can continue this way using Equation (25) to obtain that for an edge $\tau^{\prime }$ of the N-cycle scenario, we have*

$$q_{\left(c,\tau^{\prime }\right)}=\left\{\begin{array}{cc} p_{-} & \tau^{\prime }=\tau \\ p_{+} & \mathrm{otherwise}. \end{array}\right.$$

**Figure 18:**
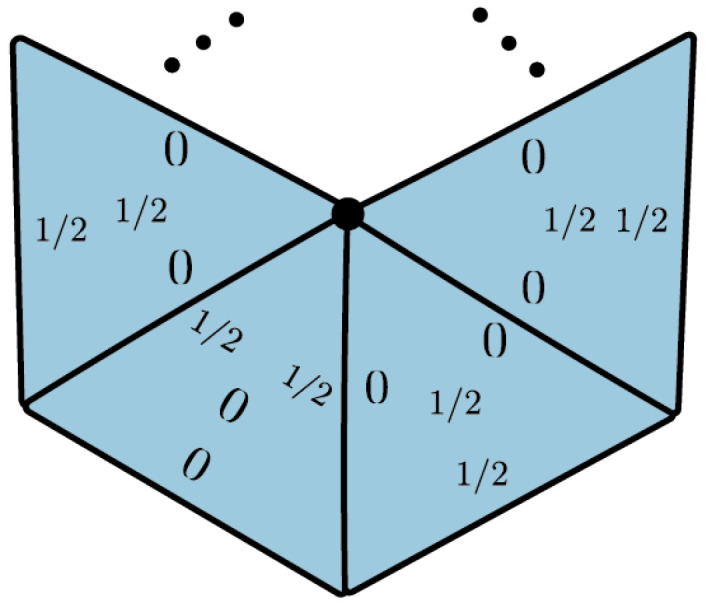
The contextual vertex obtained by the collapsing $\pi :C_{N}\to C_{1}$.

*According to Corollary 8, q is a contextual vertex in the N-cycle scenario; see Figure 18.*

The vertex detected in Example 6 generalizes the PR boxes defined in Example 3.

**Definition** **13.** *A PR box on an N-cycle scenario is a simplicial distribution $p\in \mathrm{sDist}({\tilde{C}}_{N})$, such that $p_{\sigma }=p_{\pm }$ for $\sigma \in \{\sigma_{i}:\;i=1\cdots ,N\}$ with the further restriction that the number of $p_{-}$’s is odd.*

It is clear from the definition that there are $2^{N-1}$ PR boxes on the *N*-cycle scenario. All of them can be obtained from the one given in Example 6 by the action of $\mathrm{dDist}({\tilde{C}}_{N})$ in a way similar to the CHSH scenario discussed in Example 3. Therefore, by Proposition 5, the PR boxes are contextual vertices in the cycle scenario.

Next, we describe a one-dimensional simplicial set obtained by gluing *n* copies of $C_{1}$ at their vertex. More explicitly, this simplicial set, denoted by $\vee_{i=1}^{n}C_{1}$, consists of the generating 1-simplices $\tau_{1},\cdots ,\tau_{n}$ with the identifying relations

$$d_{0}\tau_{i}=v=d_{1}\tau_{i},\;\;\;i=1,\cdots ,n,$$

where *v* is the unique vertex. The cone space $C(\vee_{i=1}^{n}C_{1})$ consists of *n* triangles given by $\left(c,\tau_{i}\right)$ where i=1,⋯,n. We consider simplicial distributions on the cone of $\vee_{i=1}^{n}C_{1}$. Such a distribution *p* is determined by the *n* distributions $p_{\left(c,\tau_{i}\right)}$ on $Z_{2}^{2}$. For convenience of notation, we will write $\left(p_{1},\cdots ,p_{n}\right)$ for this tuple of distribution, i.e., $p_{i}=p_{\left(c,\tau_{i}\right)}$.

**Proposition**  **11.** *The polytope $\mathrm{sDist}(C\left(\vee_{i=1}^{n}C_{1}\right))$ can be identified with the following subpolytope of the (n+1)-cube:*

(31) $$\left\{\left(a_{1},\cdots ,a_{n},b\right)\in {\left[0,1\right]}^{n}:\;\frac{1-a_{i}}{2}\leq b\leq \frac{1+a_{i}}{2},\;\forall 1\leq i\leq n\right\}$$

**Proof.**  The nondegenerate edges in $C(\vee_{i=1}^{n}C_{1})$ are $\tau_{1},\cdots ,\tau_{n},\left(c,v\right)$, and for every 1≤i≤n, the nondegenerate triangle $\left(c,\tau_{i}\right)$ has the edges $\left(c,v\right),\tau_{i},\left(c,v\right)$. By Proposition 4 and Corollary 7, we see that $\mathrm{sDist}(C\left(\vee_{i=1}^{n}C_{1}\right))$ can be identified with the set of $\left(a_{1},\cdots ,a_{n},b\right)\in {\left[0,1\right]}^{n}$ satisfying the following inequalities:

(32) $$\begin{array}{c} b+a_{i}+b\geq 1\\ b+a_{i}-b\leq 1\\ b-a_{i}+b\leq 1\\ -b+a_{i}+b\leq 1 \end{array}$$

for every 1≤i≤n. The set of inequalities in (32) is equivalent to $\frac{1-a_{i}}{2}\leq b\leq \frac{1+a_{i}}{2}$, 1≤i≤n. □

**Proposition**  **12.** *The polytope $\mathrm{sDist}(C\left(\vee_{i=1}^{n}C_{1}\right))$ has $2^{n}+1$ vertices:*

1. *There are two deterministic vertices given by $\left(\delta^{00},\cdots ,\delta^{00}\right)$ and $\left(\delta^{11},\cdots ,\delta^{11}\right)$.*
2. *The contextual vertices are of the form $\left(p_{1},\cdots ,p_{n}\right)$, where $p_{i}\in \left\{p_{+},p_{-}\right\}$ for every 1≤i≤n, with at least one j satisfying $p_{j}=p_{-}$.*

**Proof.**  The edge (c,v) appears twice in every nondegenerate triangle of $C(\vee_{i=1}^{n}C_{1}))$; thus, every outcome assignment s on this measurement space is determined by $s_{\left(c,v\right)}\in \left\{0,1\right\}$. Therefore, we have only $\left(\delta^{00},\cdots ,\delta^{00}\right)$ and $\left(\delta^{11},\cdots ,\delta^{11}\right)$ as deterministic distributions. Now, let us denote the polytope in (31) by *P* and find its vertices. Given an element $(a_{1},\cdots ,a_{n},b)\in P$ such that $a_{j}\notin \left\{0,1\right\}$ for some 1≤j≤n, there exists distinct $a_{j}^{\prime },a_{j}^{\prime \prime }\in \left[0,1\right]$, and 0<α<1, such that

$$\frac{1-a_{j}^{\prime }}{2}\leq q\leq \frac{1+a_{j}^{\prime }}{2},\;\;\frac{1-a_{j}^{\prime \prime }}{2}\leq q\leq \frac{1+a_{j}^{\prime \prime }}{2},\;\;\mathrm{and}\;\;\alpha a_{j}^{\prime }+\left(1-\alpha \right)a_{j}^{\prime \prime }=a_{j}$$

Therefore, we have

$$\left(a_{1},\cdots ,a_{n},q\right)=\alpha \left(a_{1},\cdots ,a_{j}^{\prime },\cdots ,a_{n},q\right)+\left(1-\alpha \right)\left(a_{1},\cdots ,a_{j}^{\prime \prime },\cdots ,a_{n},q\right)$$

We conclude that if $\left(a_{1},\cdots ,a_{n},q\right)$ is a vertex in *P*, then $a_{1},\cdots ,a_{n}\in \left\{0,1\right\}$. In the case that $a_{1}=\cdots =a_{n}=1$, we have two vertices (1,⋯,1,0) and (1,⋯,1,1). Let $f:C(\vee_{i=1}^{n}C_{1})\to P$ denote the bijection given in Proposition 11. One can see that by applying the inverse of *f*, we obtain the two deterministic vertices $\left(\delta^{00},\cdots ,\delta^{00}\right)$ and $\left(\delta^{11},\cdots ,\delta^{11}\right)$. On the other hand, if $a_{j}=0$ for some 0≤j≤1, then

$$\frac{1}{2}=\frac{1-0}{2}\leq q\leq \frac{1+0}{2}=\frac{1}{2}.$$

We obtain that $q=\frac{1}{2}$. Therefore, the rest of the vertices are of the form $\left(a_{1},\cdots ,a_{n},\frac{1}{2}\right)$, where $a_{i}\in \left\{0,1\right\}$ for every *i* and $a_{j}=0$ for at least one *j*. By applying the inverse of *f*, we obtain the desired contextual vertices. □

Our main result in this section relates a topological invariant, the fundamental group, to the number of contextual vertices. Given a one-dimensional simplicial set *X* regarded as a graph, consider a maximal tree T⊂X. The collapsing map can be applied to the edges in T to obtain a map π:X→X/T, where X/T is of the form $\vee_{i=1}^{n_{X}}C_{1}$. The number $n_{X}$ is a topological invariant of the graph that gives the noncontractible circles. This number is independent of the chosen maximal tree. The fundamental group $\pi_{1}\left(X\right)$ is defined to be the free group on the set of $n_{X}$ edges in $X_{1}^{\circ }-T_{1}^{\circ }$; see (Section 1.A in [23]).

**Theorem**  **4.** *Let X be a connected one-dimensional measurement space and $n_{X}$ denote the number of generators of the fundamental group $\pi_{1}\left(X\right)$. Then, there exists at least $\left(2^{n_{X}}-1\right)2^{|X_{0}|-1}$ contextual vertices in sDist(C(X)).*

**Proof.**  For simplicity, we will write $n=n_{X}$. Let *T* be a maximal tree in *X*. We have the collapsing map $\pi :X\to X/T=\vee_{i=1}^{n}C_{1}$. According to Corollary 8, applying ${\left(C\pi \right)}^{*}$ to the contextual vertices described in Proposition 12, we obtain contextual vertices of sDist(C(X)). First, we will show that these vertices are in different orbits under the action of sDist(C(X)). Given two different contextual vertices $\left(p_{1},\cdots ,p_{n}\right)$ and $\left(q_{1},\cdots ,q_{n}\right)$ of $\mathrm{sDist}(C\left(\vee_{i=1}^{n_{X}}C_{1}\right))$, there exists 1≤j≤n, such that $p_{j}=p_{-}$ and $q_{j}=p_{+}$. Fix one circle *C* in *X*, such that the image of ${\pi |}_{C}$ contains only $\tau_{j}$ as a nondegenerate 1-simplex (i.e, *C* collapsed to the circle generated by $\tau_{j}$). We have

$${\left(C\pi \right)}^{*}\left(p_{1},\cdots ,p_{n}\right){|}_{C(C)}=C{\left(\pi {|}_{C}\right)}^{*}\left(p_{j}\right)=C{\left(\pi {|}_{C}\right)}^{*}\left(p_{-}\right)$$

which is the PR box of Example 6. On the other hand, one can see using the same technique of Example 6 that the restriction of ${\left(C\pi \right)}^{*}\left(q_{1},\cdots ,q_{n}\right)$ to C(C) is the noncontextual distribution $e_{+}$, the identity element of $G_{\pm }\left(C(X)\right)$ (see Definition 7). Therefore, these two restrictions are not in the same orbits under the action of sDist(C(C)). We conclude that ${\left(C\pi \right)}^{*}\left(p_{1},\cdots ,p_{n}\right)$ and ${\left(C\pi \right)}^{*}\left(q_{1},\cdots ,q_{n}\right)$ are not in the same orbit under the action of sDist(C(X)). So far, we have proved that there are $2^{n}-1$ contextual vertices in sDist(C(X)) that lie in different orbits. Observe that every such vertex has $p_{\pm }$ on every nondegenerate triangle of C(X); thus, the only two outcome assignments that fix this vertex are those that restrict to $\delta^{00}$ on every nondegenerate triangle or $\delta^{11}$ on every nondegenerate triangle. We conclude that the orbit of such a vertex has $\frac{\left|\mathrm{dDist}(C(X))\right|}{2}=\frac{2^{|X_{0}|}}{2}=2^{|X_{0}|-1}$ elements. By Proposition 5, all these distributions are contextual vertices. □

**Corollary** **9.** *A simplicial distribution in the group $G_{\pm }\left(CX\right)$ (Proposition 6) is noncontextual if and only if it belongs to the subgroup*

$$e_{+}\cdot \mathrm{dDist}\left(C\left(X\right)\right)=\{e_{+}\cdot \delta^{s}:\;\delta^{s}\in \mathrm{dDist}\left(C(X)\right)\}.$$

**Proof.**  The element $e_{+}$ is noncontextual since we have

$$e_{+}=\frac{1}{2}\delta^{t}+\frac{1}{2}\delta^{r}$$

where $t_{\left(c,\tau \right)}=\left(0,0\right)$ and $r_{\left(c,\tau \right)}=\left(1,0\right)$ for every $\tau \in X_{1}^{\circ }$, and ${\left(e_{+}\cdot \delta^{s}\right)}_{\sigma }=p_{\pm }$ for every nondegenerate 2-simplex *σ* of C(X). Therefore, the coset $e_{+}\cdot \mathrm{dDist}\left(C\left(X\right)\right)$ is a subset of $G_{\pm }\left(CX\right)$, and all its elements are noncontextual. Moreover, since sDist(C(X)) is a commutative monoid and $e_{+}\cdot e_{+}=e_{+}$, the subset $e_{+}\cdot \mathrm{dDist}\left(C\left(X\right)\right)$ is in fact a subgroup of $G_{\pm }\left(CX\right)$.

To conclude that the remaining distributions are all contextual, we will use Theorem 4. For a one-dimensional (connected) simplicial set *X*, the Euler characteristic [23] is given by $\chi \left(X\right)=1-n_{X}$. Alternatively, it can be computed using the formula

$$\chi \left(X\right)=|X_{0}\left|-\right|X_{1}^{\circ }|.$$

Therefore, we have $n_{X}=|X_{1}^{\circ }\left|-\right|X_{0}|+1$. Using this, we find that the number of contextual vertices detected in Theorem 4 is equal to $2^{|X_{1}^{\circ }|}-2^{|X_{0}|-1}$. On the other hand, we have

$$|G_{\pm }C(X)|-|e_{+}\cdot \mathrm{dDist}C(X)|=2^{|X_{1}^{\circ }|}-2^{|X_{0}|-1}$$

where we used the fact that the size of the coset is half the size of dDist(C(X)) since it is the orbit of $e_{+}$. Therefore, the contextual vertices detected in Theorem 4 are precisely those distributed in $G_{\pm }\left(CX\right)-\left(e_{+}\cdot \mathrm{dDist}\left(CX\right)\right)$. □

**Example**  **7.** *The Bell scenario (m,n,2,2) is represented by the complete bipartite graph $K_{m,n}$. This graph has m+n vertices and m·n edges. Therefore, by Theorem 4, we have at least $2^{mn}-2^{m+n-1}$ contextual vertices in the scenario (m,n,2,2).*

### 5.3. Contextual Vertices of the Cycle Scenario

We conclude this section by showing that PR boxes constitute all the contextual vertices in the cycle scenario using the collapsing method. Let us set $X={\tilde{C}}_{N}$. The polytope $P_{X}$ associated to the cycle scenario has dimension $R^{2N}$. This is a consequence of Proposition 4, since the number of nondegenerate edges of the *N*-cycle space is 2N. Following Figure 9, the edges on the boundary of the *N*-cycle space will be denoted $\tau_{i}$, while interior edges will be denoted $x_{i}$, where i=1,⋯,N.

**Lemma**  **11.** *Let p be a simplicial distribution on the N-cycle scenario, such that $p_{x_{i}}$ is deterministic for some 1≤i≤N. Then, p is noncontextual.*

**Proof.**  Assume that $p_{x_{i}}=\delta^{a}$ for some *a*. Let *q* be the deterministic distribution given by $q_{\sigma_{j}}=\delta^{00}$ for all 1≤j≤N distinct from *i* and $q_{\sigma_{i}}=\delta^{11}$. Since ${\left(q\cdot p\right)}_{x_{i}}=\delta^{0}$ and *p* is noncontextual if and only if q·p is noncontextual, we can assume that a=0. Let $\bar{X}$ denote the quotient *X* obtained by collapsing $x_{i}$. The resulting space $\bar{X}$ is a classical *N*-disk. Consider the map

$$\pi^{*}:\mathrm{sDist}\left(\bar{X}\right)\to \mathrm{sDist}\left(X\right)$$

induced by the quotient map $\pi :X\to \bar{X}$. There exists a simplicial distribution $\tilde{p}$ on the classical *N*-disk, such that $\pi^{*}\left(\tilde{p}\right)=p$. Since every distribution on the *N*-disk is noncontextual, *p* is noncontextual. □

**Proposition** **13.** *Contextual vertices of the polytope of simplicial distributions on the N-cycle scenario are given by the PR boxes.*

**Proof.**  By Lemma 11, for a vertex *p*, there cannot be a deterministic edge on any of the $x_{i}$’s. By Corollary 3, *p* is a vertex if and only if rank(p)=2N. Therefore, there are precisely *N* deterministic edges $z_{1},\cdots ,z_{N}$, all of which lie on the boundary. The distribution $p_{\sigma_{i}}$, which is given by $p_{\pm }$, for each triangle, has rank 2. Let (A,b) be as in Corollary 2, so that $P_{X}=P\left(A,b\right)$. Let *p* be the distribution, such that ${p|}_{\sigma_{i}}=p_{\pm }$ for every $\sigma_{i}$, and let ${Ƶ}_{p}$ index the inequalities tight at *p*. There are 2N such tight inequalities, since there are *N* nondegenerate simplices $\sigma_{i}$ and each corresponding distribution $p_{\sigma_{i}}$ has two zeros; see Figure 18. Denoting $A\left[{Ƶ}_{p}\right]:=A_{p}$, we order the columns of $A_{p}$ by $x_{1}\cdots ,x_{N},\tau_{1},\cdots ,\tau_{N}$. Up to elementary row operations, we have

$$A_{p}=\left(\begin{array}{cc} E & 0\\ 0 & I \end{array}\right),$$

where 0 is an N×N matrix of zeros and *I* is the N×N identity matrix. Then $\mathrm{rank}\left(A_{p}\right)=N+\mathrm{rank}\left(E\right)$. Multiplying each row of *E* by −1, if necessary, we can write

$$E=\left(\begin{array}{ccccc} 1 & {\left(-1\right)}^{c_{1}+1} & 0 & \cdots & 0\\ 0 & 1 & {\left(-1\right)}^{c_{2}+1} & \cdots & 0\\ \vdots & \vdots & \ddots & \vdots & \vdots \\ 0 & \cdots & 0 & 1 & {\left(-1\right)}^{c_{N}+1}\\ {\left(-1\right)}^{c_{1}+1} & 0 & \cdots & 0 & 1 \end{array}\right)$$

Let us define $c=\sum_{i=1}^{N}c_{i}\;\mathrm{mod}\;2$. Then, rank(E)=N if c=1, otherwise rank(E)=N−1. In the former case, we have $p_{x_{i}}^{0}=1-p_{x_{i}}^{0}$, which implies that $p_{x_{i}}^{0}=1/2$. Hence, $p_{\sigma_{i}}$s are all given by $p_{\pm }$. The condition that c=1 implies that the number of $\sigma_{i}$’s with $p_{\sigma_{i}}=p_{-}$ is odd. □

Two vertices $v,v^{\prime }$ of a full-dimensional polytope $P\subset R^{d}$ are called neighbors if $\mathrm{rank}(A\left[{Ƶ}_{v}\cap {Ƶ}_{v^{\prime }}\right])=d-1$.

**Corollary** **10.** *All neighbors of a PR box are deterministic distributions.*

**Proof.**  A PR box *p* corresponds to a nondegenerate vertex, meaning that the number 2N of tight inequalities is precisely the dimension of the polytope. One property of nondegenerate vertices is that if ${Ƶ}_{p}$ indexes the tight inequalities of a PR box *p*, and *Ƶ* is a set differing from ${Ƶ}_{p}$ by one element, then $p^{\prime }=A{\left[Ƶ\right]}^{-1}b$ is also a vertex, so long as it satisfies the remaining inequalities. In this case, *p* and $p^{\prime }$ are neighbors. A neighbor is obtained by replacing a tight inequality with another, which amounts to replacing one zero with another. Doing so will make one of the $x_{i}$s a deterministic edge. Lemma 11 implies that $p^{\prime }$, if it is a vertex of $P_{X}$, is noncontextual, and thus a deterministic distribution. □

### 5.4. Conclusions

In this paper, we demonstrate novel techniques from the theory of simplicial distributions introduced in [7]. We present topological proofs for the sufficiency of the circle inequalities for the noncontextuality of distributions on the cycle scenario. This proof extends the topological proof of the CHSH scenario in [7]. We go beyond the cycle scenarios and study the flower scenario depicted in Figure 1 that generalizes the bipartite Bell scenarios consisting of 2 measurements for Alice and *m* measurements for Bob. Our main insight in the proof is the topological interpretation of Fourier–Motzkin elimination and the gluing and extension methods of distributions on spaces. We also explore two new features of scenarios available in the simplicial setting: (1) collapsing measurement spaces to detect contextual vertices; and (2) applying the monoid structure of simplicial distributions to generate vertices. An appealing feature of the collapsing technique featured here is that previously unknown types of Bell inequalities can be discovered from those that are known; see Section 5.1. These Bell inequalities may have desirable properties, such as having quantum violations that are more robust to noise, which may be of both theoretical and practical interest.
